# The roles and mechanisms of natural compounds in preventing and treating viral reproductive disorders in major economic livestock

**DOI:** 10.1080/21505594.2026.2723577

**Published:** 2026-08-24

**Authors:** Qiling Su, Jiang Wu, Qinyao Shi, Haien Chen, Chongkai Hu, Yidan Huang, Kai Kang

**Affiliations:** College of Coastal Agricultural Sciences, Guangdong Ocean University, Zhanjiang, China

**Keywords:** Natural compounds, prevention and control, major economic livestock, viral reproductive disorders, action and mechanism

## Abstract

Reproductive disorders from viral infections cause major economic losses in livestock. This review provides a comprehensive mapping of natural compounds (monomers, extracts, and traditional Chinese medicine formulas) against 12 key viruses causing reproductive disorders in pigs, cattle, and sheep. A total of 297 independent studies were included. The compounds targeted multiple nodes within four conserved host signaling networks: NF-κB (TLR4, p65, IRF3, IκBα, ERK1/2), MAPK (p38 MAPK, JNK), apoptosis-related factors (Caspase-3, Bcl-2, Bax), and autophagy-related factors (AKT, p62). By modulating these networks, this multi-target, on-demand regulatory strategy endows natural compounds with broad-spectrum regulatory effects on host responses and the ability to reduce drug resistance. These findings consequently provide a theoretical basis for developing a single natural product combination that is effective against multiple reproductive disorder viruses.

## Introduction

Historically, virus-induced reproductive disorders have posed persistent threats to the health and productivity of major economic livestock species and remain a serious challenge to the global farming industry. Globally, these viral reproductive disorders cause enormous economic losses each year. Pathogens such as African swine fever virus (ASFV) and bovine viral diarrhea virus (BVDV) severely impair animal reproductive performance, leading to embryonic death, abortion, stillbirth, and infertility. The direct economic losses caused by BVDV are alarming: in a survey across 15 countries, losses per animal reached as high as $687.80, and the average direct loss per newborn dairy cow exceeded that per beef cow [1]. Porcine circovirus type 2 (PCV2) has caused annual economic losses exceeding 2 billion US dollars to the global pig industry [2]. Furthermore, classical swine fever virus (CSFV) and porcine reproductive and respiratory syndrome virus (PRRSV) impose substantial economic burdens. The CSFV outbreak that occurred in the Netherlands in 1997–1998 resulted in a single-event financial impact of as much as 2.3 billion US dollars [3]. In Australia, an epidemic of CSFV or PRRSV can result in regional income losses of approximately 10 to 30 million Australian dollars, based solely on sales losses and disposal costs, and can lead to an annual reduction of up to 11% in the nation’s total pig income [4]. Simulation studies of foot-and-mouth disease virus (FMDV) have indicated that if the detection of an outbreak in California, USA, is delayed by 7 to 22 days, the resulting national agricultural welfare losses could range from 2.3 billion to 69 billion US dollars [5]. Between 2016 and 2020, porcine reproductive and respiratory syndrome virus (PRRSV) infections in the United States resulted in annual economic losses of approximately $1.2 billion [6]. Collectively, these data indicate that viral diseases characterized by reproductive disorders pose a major economic threat to intensive farming systems worldwide. The reproductive performance of pigs, cattle, and sheep, which are globally important economic livestock species, is directly linked to the output value of animal husbandry and food security. These viral disorders have thus become critical bottlenecks for industry development.

Current antiviral control strategies rely heavily on isolation, culling, supportive therapies, and vaccination. However, these approaches face significant limitations. Isolation and culling are economically burdensome and not always feasible in resource-limited settings. Taking ASFV as an example, the outbreak in China between August 2018 and July 2019 resulted in an estimated 43.46 million pigs dying or being culled because of infection or control measures, contributing to total economic losses of approximately US$111.2 billion, which was 0.78% of China’s GDP in 2019 [7]. Supportive therapies alleviate clinical symptoms without eliminating the virus. Vaccination, while widely used, is often compromised by the high genetic diversity and continuous mutation of viruses, resulting in insufficient cross-protection, vaccine breakdown, and even reversal to virulence. For example, in multiple pig farms in China that had been vaccinated with the C-strain vaccine, outbreaks of CSFV subtype 2.1d still caused significant economic losses [8]. Furthermore, some vaccines may cause immunosuppression or interfere with diagnostic surveillance. As a result, culling and supportive therapies have become the default options, increasing the economic and operational burdens on farmers. In this context, natural compounds have demonstrated unique advantages in antiviral research because of their broad-spectrum biological activities and low toxicity profiles [9]. Secondary metabolites of plant origin such as flavonoids, terpenoids, coumarins, and saponins; polysaccharide compounds such as chitosan; and natural products derived from marine and microbial sources have been shown to inhibit viral replication and interfere with the viral life cycle [10–14]. Traditional Chinese medicine compounds and their active constituents have emerged as valuable resources for antiviral drug development because of their multitarget mechanisms, low resistance potential, and synergistic effects. This review provides a comprehensive and systematic summary of the effects of these natural compounds against viral reproductive disorders in pigs, cattle, and sheep, along with their underlying mechanisms. By analyzing their common mechanisms, which include targeting the nuclear factor kappa-B (NF-κB) and mitogen-activated protein kinase (MAPK) signaling pathways and modulating autophagy and apoptosis, this work not only facilitates the screening and development of efficient, broad-spectrum natural antiviral agents but also offers new prospects for the development of a green and sustainable animal husbandry industry. A list of abbreviations is provided in Supplementary Table S1.

## Information sources and search strategy

On 18 November 2025, a systematic literature search was performed in the Web of Science, PubMed, and Science Direct databases. Preliminary comparisons revealed extremely high duplication rates among the three databases, with most eligible studies in Web of Science and Science Direct already indexed in PubMed, and no notable number of unique records was identified. Therefore, this study ultimately used only the PubMed database as the source of literature for subsequent screening and analysis. Notably, restricting the search to PubMed alone is a limitation of this systematic review, as the possibility of relevant studies being present in other databases (such as Scopus, Cochrane Library, and Embase) cannot be completely excluded. Future reviews should extend the search to a broader range of databases.

During the retrieval and screening phase, this systematic review was conducted in accordance with the PRISMA guidelines. The completed PRISMA checklist is provided in Supplementary File S1. Two authors independently searched the PubMed database for relevant literature on 12 important animal viruses using the following keyword combinations: (ASFV) OR (African Swine Fever Virus); (CSFV) OR (Classical Swine Fever Virus); (FMDV) OR (Foot‑and‑Mouth Disease Virus); (JEV) OR (Japanese Encephalitis Virus); (PCV2) OR (Porcine Circovirus Type 2); ((PPV) OR (Porcine Parvovirus)) AND (anti); (PRRSV) OR (Porcine Reproductive and Respiratory Syndrome Virus); (PRV) OR (Pseudorabies Virus); (BoHV‑1) OR (Bovine Herpesvirus 1); (BVDV) OR (Bovine Viral Diarrhea Virus); ((CpHV‑1) OR (Caprine Herpesvirus 1)) AND (anti); and (BDV) OR (Border Disease Virus). An initial set of 42,945 records was obtained. After 3,700 duplicates were removed, 39,245 unique articles remained. After title/abstract screening, 38,864 articles were excluded, leaving 381 articles for full‑text assessment. After full‑text review, 84 articles were excluded because they were non–natural product studies, review articles, or purely in silico experiments, resulting in the preliminary inclusion of 299 studies. The detailed search strategy, including virus-specific search queries and the number of records initially identified, is documented in Supplementary Table S2.

**Figure 1.**
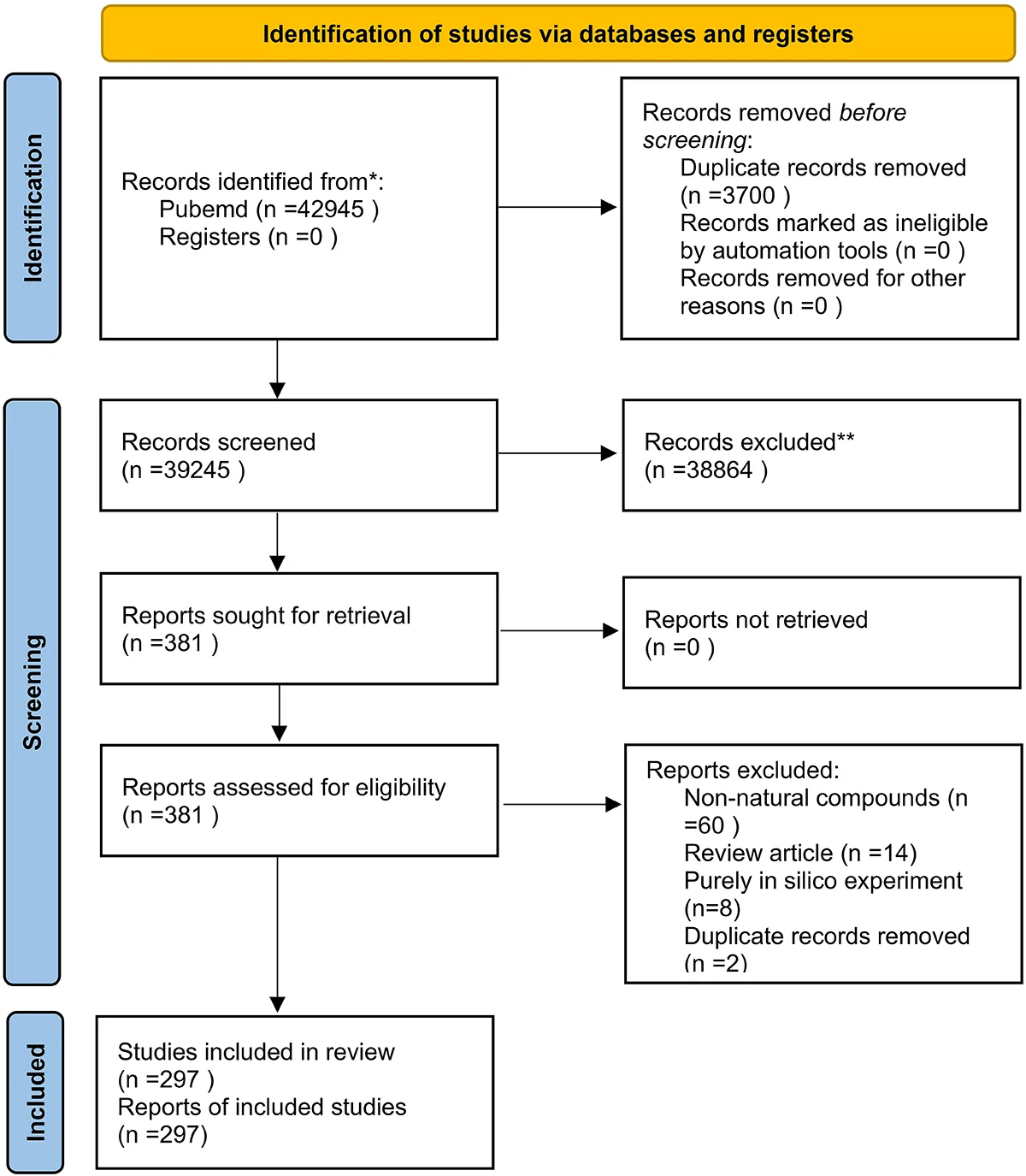
PRISMA flow diagram.

The number of included studies and their experimental type distributions for each virus were as follows: ASFV: 27 studies (25 *in vitro*, 2 combined *in vitro* and *in vivo*); CSFV: 10 studies (5 *in vitro*, 5 *in vivo*); FMDV: 12 studies (9 *in vitro*, 3 *in vivo*); JEV: 39 studies (20 *in vitro*, 19 combined *in vitro* and *in vivo*); PCV2: 39 studies (20 *in vitro*, 9 combined *in vitro* and *in vivo*, 10 *in vivo*); PPV: 4 studies (2 *in vitro*, 1 *in vivo*, 1 combined); PRRSV: 73 studies (57 *in vitro*, 10 combined, 6 *in vivo*); PRV: 49 studies (27 *in vitro*, 20 combined, 2 *in vivo*); BDV: 0 studies; BoHV‑1: 13 studies (all *in vitro*); BVDV: 30 studies (18 *in vitro*, 12 combined); and CpHV‑1: 3 studies (2 *in vitro*, 1 *in vivo*). Among the above counts, two studies were double-counted because they involved multiple viruses. After deduplication, a total of 297 independent studies were ultimately included in this systematic review. The study selection process followed the PRISMA flow diagram (Figure 1). Given the exploratory nature of most included studies and the heterogeneity in experimental designs, a formal risk-of-bias assessment and GRADE evidence grading were not performed. This is inherent to a scoping review aiming to map mechanistic pathways rather than to quantitatively synthesize clinical efficacy. Readers should interpret the findings as hypothesis-generating rather than confirmatory.

During the data extraction and analysis phase, a stratified approach was adopted. For viruses with fewer than 25 included studies (CSFV, FMDV, PPV, BoHV‑1, and CpHV‑1), a detailed analysis of both mixtures and monomers is presented. For viruses with 25 or more included studies (ASFV, JEV, PCV2, PRRSV, PRV, and BVDV), information on monomers is summarized in tabular form, while studies on crude extracts are reviewed in the main text. Furthermore, a cross-virus analysis was conducted to identify targets shared by four or more viruses, with emphasis on potential broad-spectrum antiviral lead compounds.

## Causes of reproductive disorders in major economic livestock

**Figure 2.**
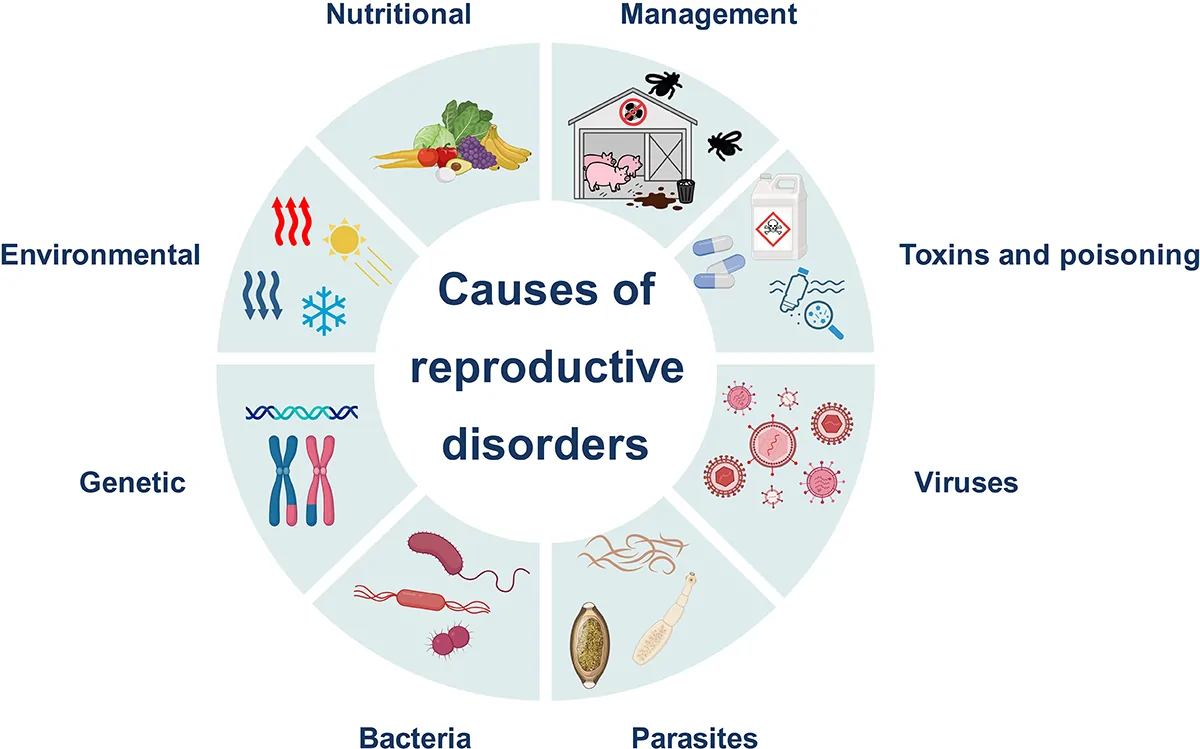
Factors that contribute to reproductive disorders in mammals. Drawn by BioRender.

Within the global agricultural system, pigs, cattle, and sheep, as the most crucial livestock species, serve as primary sources of essential animal products such as meat, milk, and wool. These animals are key drivers of the agricultural economy and are crucial for safeguarding the food supply. However, reproductive disorders significantly affect the output of animal products. The causes of these disorders can be broadly categorized into noninfectious and infectious factors. Noninfectious factors include the environment, management practices, nutrition, genetics, toxin exposure, and poisoning. Infectious factors primarily include viruses, bacteria, and parasites (Figure 2). Among infectious factors, viruses targeting the reproductive system often induce large-scale reproductive disorders, posing severe threats to the sustainable development of the animal husbandry industry.

### Noninfectious factors leading to reproductive disorders in livestock

Environmental factors such as high temperatures adversely affect the endocrine, neural, and immune systems of animals [15]. These disruptions may subsequently interfere with gametogenesis, fertilization processes, and early embryonic development [16,17], ultimately leading to estrous abnormalities [18], management deficiencies [19] and nutritional imbalances [20]. Genetically, single-gene defects may lead to infertility by disrupting the regulatory mechanisms of the hypothalamic-pituitary-gonadal axis [21]. In addition, exposure to toxins, such as heavy metals, can occur [22]. These noninfectious factors are not the primary focus of this review and are therefore only briefly noted here.

### Infectious factors leading to reproductive disorders in livestock

Among infectious factors, viral infections are important causes of reproductive disorders in mammals. Viruses can severely reduce semen quality through seminal infection [23]. Furthermore, they can infect the female reproductive system or be transmitted vertically, leading to abortion, stillbirth, infertility, or the production of weak offspring in dams, thereby decreasing herd reproductive rates [24]. Nonspecific bacterial infections, such as uterine infections, are most commonly associated with the presence of *Escherichia coli*, *Trueperella pyogenes*, *Fusobacterium necrophorum*, Prevotella species, or Bacteroides species, which can induce endometritis and subsequently impair the reproductive function of dams [25]. Parasitic infections, such as those caused by *Toxoplasma gondii*, can directly impair reproductive processes through genital tract inflammation or immune-mediated damage.

## Viruses causing reproductive disorders in major economic livestock

The viruses responsible for porcine reproductive failure include primarily ASFV, CSFV, FMDV, JEV, PCV2, PPV, PRRSV, and PRV. ASFV is highly stable in the environment and can remain viable in frozen meat products for several years. It can be readily transmitted over long distances through contaminated pork products. This virus is extremely pathogenic to both domestic and wild pigs, with acute infections resulting in a case fatality rate of 100%. Currently, no safe and effective commercial vaccines for ASFV are available, making disease control largely dependent on stringent biosecurity measures and the culling of infected animals [26]. ASFV, CSFV, and FMDV are notifiable diseases listed by the World Organization for Animal Health. CSFV infection can be effectively controlled by highly efficacious vaccines, which have enabled successful eradication of CSFV in many countries. However, outbreaks still lead to stamping-out policies and substantial trade restrictions [27]. FMDV is highly contagious; although it has a low mortality rate in adult pigs, it causes abortion in sows and growth retardation in young pigs. Its occurrence often triggers trade embargoes, resulting in substantial economic losses [28]. Pigs serve as key amplifying hosts for JEV. The virus circulates in pigs, infecting increasing numbers of mosquitoes, which then transmit it to humans and other animals; it causes reproductive failure in pigs and severe encephalitis in humans [29]. PCV2 is endemic to swine populations worldwide. It induces immunosuppression, increasing the susceptibility of pigs to secondary infections and contributing to a range of PCV2-associated diseases. PPV is highly resistant to environmental conditions. While infection is often subclinical, it primarily affects first-litter sows, causing reproductive failure. Vaccination is effective at controlling both PCV2 and PPV infections [30,31]. PRRSV is difficult to eradicate because of its ability to cause immunosuppression and its high genetic variability and rate of recombination. These characteristics pose major challenges for vaccine development and disease control. Following infection, PRV establishes lifelong latency in the neural ganglia. Viral reactivation and shedding can occur under stress. Many countries have implemented eradication programs using gene-deleted vaccines accompanied by differential diagnostic tests [32].

The primary viruses associated with bovine reproductive disorders include BoHV-1 and BVDV. Following initial infection, BoHV-1 establishes lifelong latency in sensory ganglia. Viral reactivation and shedding can be triggered by stress or the administration of corticosteroids, and they constitute the underlying cause of recurrent disease. Recently, gene-deleted marker vaccines (such as gE-deleted vaccines) have become available for immunization [33,34]. BVDV primarily infects cattle, with persistently infected animals being the most important source of infection. These animals shed the virus throughout their lifetime via secretions, excretions, and semen and can also transmit the virus vertically across the placenta, leading to fetal infection [35]. The presence of persistently infected animals serves as the central driving force for the long-term maintenance and transmission of BVDV within a herd. A single persistently infected animal in a population is sufficient to initiate continuous transmission among susceptible individuals and results in recurrent reproductive disorders. Experimental data indicate that under high-density conditions, one persistently infected calf infected all 10 contact animals within 30 days; under low-density conditions, one persistently infected calf infected 77% of 48 contact animals within 100 days [36]. These findings indicate that continuous viral shedding from persistently infected animals leads to efficient transmission and that high-density housing significantly accelerates the spread of infection. Furthermore, economic analyses of direct exposure to persistently infected animals have estimated the associated losses at 5.26 per head for mortality and 88.26 per head for production performance [37].

The viruses primarily responsible for reproductive disorders in sheep and goats include BDV and CpHV-1. BDV is an important pathogen that causes reproductive failure in both sheep and goats. Its impact is similar to that of BVDV; infection during early gestation can lead to the birth of persistently infected lambs. These lambs, being immunotolerant, carry and shed the virus throughout their lives, thus serving as a major source of transmission. Control strategies for BDV can draw on those established for BVDV, with the core approach being the identification and culling of persistently infected animals. In some regions, vaccines with cross-protective efficacy may also be used for immunization. CpHV-1 specifically infects goats, and its pathogenic mechanism closely resembles that of BoHV-1. Inactivated or gene-deleted marker vaccines for CpHV-1 are available. Immunizing healthy herds, particularly replacement ewes, can effectively prevent viral replication and the establishment of latent infection, thereby helping to control reproductive disorders.

These viruses disrupt the reproductive physiology of the host through complex pathogenic mechanisms, such as vertical transmission, colonization of the reproductive tract, and placental infection, leading to endocrine disruption, damage to germ cells, and impaired embryonic development, among other adverse outcomes (Table 1). A thorough understanding of the pathogenic mechanisms and epidemiological characteristics of these viruses is essential for formulating precise prevention and control strategies, which are crucial for ensuring stable production and economic sustainability in the livestock industry. In this context, the search for natural compounds with antiviral activity has emerged as a viable approach for the routine control of viral infection and transmission, offering considerable potential for promoting sustainable development in the animal husbandry sector.

**Table 1.** Basic information on reproductive disorder viruses in pigs, cattle, and sheep.

Primary host	Virus	Family/genus	Nucleic acid type	Genotyping/serotyping	Epidemic area	Transmission route	Clinical symptom	Pathological changes	Cite
Pig	African Swine Fever Virus	*Asfarviridae*/*Asfivirus*	Double-stranded DNA	24 genotypes; 8 serogroups	Africa, Europe, Asia	Direct contact, Indirect contact (fomites), Vector-borne (soft ticks of the genus *Ornithodoros*)	Abortion in pregnant sows, stillbirths and reduced litter size, high fever, skin hyperemia/cyanosis, hemorrhagic diarrhea, emaciation, joint swelling, intermittent fever	Placental hemorrhage, endometritis, testicular vasculitis, sperm malformation, splenomegaly and hemorrhage, hemorrhagic necrosis of lymph nodes, multifocal hemorrhages in various organs, pleuritis, pneumonia, joint hyperplasia	[38]
	Classical Swine Fever Virus	*Flaviviridae*/*Pestivirus*	Single-stranded RNA	Genotypes 1, 2, 3	Asia, Africa, South America	Oronasal route, Direct contact, Vertical transmission	Abortion and stillbirth in pregnant sows, weak neonates and congenital tremors, high fever, cutaneous petechiae, diarrhea, motor dysfunction	Placental edema and diffuse hyperemia, fetal hydrocephalus, hemorrhagic infarction of the spleen	[39]
	Foot-and-Mouth Disease Virus	*Picornaviridae*/*Aphthovirus*	Single-stranded RNA	7 serotypes (O, A, C, Asia1, SAT1-SAT3))	Africa, Asia, South America	Contact, Aerosol, Fomites	Abortion in dams, reduced semen quality in boars, vesicles on mouth/feet, salivation, lameness, myocarditis in young animals	Endometritis, orchitis, lytic necrosis of epithelial cells, striped myocardial necrosis (“tiger heart”)	[40]
	JapaneseEncephalitis Virus	*Flaviviridae*/*Flavivirus*	Single-stranded RNA	5 genotypes (I-V)	Asia, Africa, Oceania	Mosquito-borne, Vertical transmission	High fever,encephalitis, orchitis, abortion in sows	Cerebral edema, neuronal necrosis, fetal hydrops	[41,42]
	Porcine Circovirus Type 2	*Circoviridae*/*Circovirus*	Single-stranded DNA	PCV2a, PCV2b, PCV2c, PCV2d	Worldwide	Contact, Vertical transmission	Abortion, stillbirth, mummified fetuses, wasting, dyspnea, pallor	Lymphohistiocytic myocarditis in fetuses, placental vasculitis, prolonged seminal shedding	[43]
	Porcine Parvovirus	*Parvoviridae*/*Protoparvovirus*	Single-stranded DNA	Single serotype (novel genotypes exist)	Worldwide	Vertical transmission, Contact	Anestrus, infertility, mummified fetuses	Fetal cerebellar hypoplasia, placental mineralization, endometritis	[44]
	PorcineReproductive and Respiratory Syndrome Virus	*Arteriviridae*/*Porartevirus*	Single-stranded RNA	Type 1 (European), Type 2 (North American)	Worldwide	Contact, Aerosol, Semen	Abortion and stillbirth in sows, respiratory distress in piglets, high fever	Placental necrosis, interstitial pneumonia, lymphadenopathy	[45]
	Pseudorabies Virus	*Herpesviridae*/*Varicellovirus*	Double-stranded DNA	Type I/II/III (classical/variant) or 5 genotypes based on *UL44*	Asia, Eastern Europe, Latin America, Africa	Direct contact, Vertical transmission	Abortion, stillbirth, fever, vomiting, tremors, paralysis in piglets	Endometritis, placentitis, nonsuppurative encephalomyelitis/myelitis, intranuclear inclusion bodies in neurons, vascular cuffing, pulmonary edema, necrotic foci in liver/spleen, lymph node hemorrhage	[46]
Cattle	Bovine Herpesvirus 1	*Herpesviridae*/*Varicellovirus*	Double-stranded DNA	BoHV-1.1(respiratory), BoHV-1.2 (genital)	Worldwide (free in somecountries)	Respiratory/genital secretions, Vertical transmission	Abortion, infectious pustular vulvovaginitis/balanoposthitis, rhinotracheitis, high fever	Placental necrosis, respiratory mucosal ulceration, suppurative meningitis	[47]
	Bovine Viral Diarrhea Virus	*Flaviviridae*/*Pestivirus*	Single-stranded RNA	BVDV-1 (1a-1 v), BVDV-2 (2a-2d), HoBiPeV (A-D)	Worldwide (primarily Europe, Americas, Asia)	Secretions/excretions, Vertical transmission	Reduced conception rate, abortion, mucosal ulcers, diarrhea, immunosuppression	Ovarian dysfunction, early embryonic death, fetal malformations, gastrointestinal erosion, lymphoid tissue necrosis	[48]
Sheep	Border Disease Virus	*Flaviviridae*/*Pestivirus*	Single-stranded RNA	At least 7 genotypes	Europe, Australia, Asia	Vertical transmission, Direct contact	Abortion, “hairy shaker” lambs, stunted growth	Fetal cerebellar hypoplasia, hypomyelination, placentitis, production of persistently infected offspring	[49]
	Caprine Herpesvirus 1	*Herpesviridae*/*Varicellovirus*	Double-stranded DNA	Single serotype	Worldwide	Contact (nasal, genital), Vertical transmission	Vulvovaginitis/balanoposthitis in adults, fever, dyspnea, diarrhea in kids	Endometritis, placentitis, ulceration of digestive mucosa, hepatic necrosis, respiratory inflammation	[50]

## Antiviral effects of natural compounds

### Effects on viral reproductive disorders in pigs

#### Effects of natural compounds against ASFV

This section summarizes natural compounds with reported anti‑ASFV activity and their proposed antiviral mechanisms. However, most of the compounds discussed here are crude extracts or mixtures that lack well‑defined monomeric components. This absence of chemical characterization severely limits their pharmacological reproducibility, batch‑to‑batch consistency, and potential for clinical translation, as the actual active ingredients remain unknown. The compounds discussed include λ-carrageenan; a natural essential oil blend formulation containing *Eucalyptus globulus* (*E. globulus*), *Pinus sylvestris* (*P. sylvestris*), and *Lavandula latifolia* (*L. latifolia*) essential oils; peppermint extract; marine microalgae; arctiin; genistein; and benzylisoquinoline alkaloids.

##### Direct inactivation

A natural essential oil blend formulation containing *E. globulus*, *P. sylvestris*, and *L. latifolia* essential oils, as well as peppermint extract, exhibits a strong virucidal effect against ASFV, acting directly on the viral particles themselves and thereby completely inactivating the virus [51,52]. *L. latifolia* essential oils consist primarily of monoterpenoids and sesquiterpenoids. The antiviral mechanism may involve increase in the fluidity and permeability of the cell membrane induced by monoterpenes, altering the arrangement of membrane proteins, and thereby interfering with the structure of the viral envelope protein [53].

##### Attachment and entry

Prior to ASFV invasion, λ-carrageenan can directly block viral attachment [54]. Extracts from the marine microalgae *Porphyridium cruentum*, *Chlorella autotrophica*, and *Ellipsoidon* sp. can inhibit viral penetration in a dose-dependent manner and affect the attachment stage [55]. However, these inhibitory effects are not directly correlated with the sulfated polysaccharide content; the active components remain unidentified; and the low purity of the extract raises concerns regarding the feasibility of practical applications.

##### Replication

After cellular entry, the *Bacillus subtilis* metabolites arctiin and genistein can specifically bind to the adenosine triphosphate (ATP)-binding domain of ASFV topoisomerase II, directly inhibiting viral DNA replication [56]. Moreover, oral administration in pigs (2 mg/kg) reduced mortality, tissue viral loads, and pathological damage. However, investigations of this target have relied primarily on indirect approaches, including molecular docking and *in vitro* enzyme activity assays, to explore the underlying mechanism, and direct evidence from active biological systems is lacking. If the virus has completed early replication, benzylisoquinoline alkaloids can interfere with endosome/lysosome function to block viral internalization and disrupt DNA synthesis and viral factory formation [57].

In contrast, studies on compounds with clearly identified monomeric structures (such as aloe-emodin, aloperine, and berbamine) offer clear structural characterization, facilitate the elucidation of their mechanisms of action, and increase the reproducibility and translational value of research findings. Therefore, they are summarized separately in Table 2. In total, 22 antiviral compounds with clearly identified monomeric structures and their corresponding mechanisms of action are detailed in Table 2.

**Table 2.** Natural active ingredients with anti-ASFV activity.

Name	Molecular Formula	Molecular Structure	Molecular Weight (g/mol)	Experimental Model (Cell/Animal)	Tested Range	Virus Strain	Mechanism of Action	Cite
Aloe-emodin	C_15_H_10_O_5_	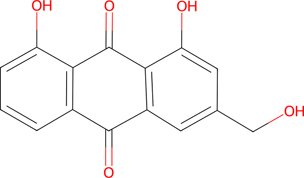	270.24	Pams	*in vitro*	ASFV strain GZ201801	Aloe-emodin significantly inhibits ASFV replication in porcine alveolar macrophages by suppressing the ASFV-activated NF-κB pathway (downregulating MyD88, p-p65, and pIκB) and inducing apoptosis (upregulating cleaved-caspase-3 and Bax; downregulating Bcl-2).	[58]
Aloperine	C_15_H_24_N_2_	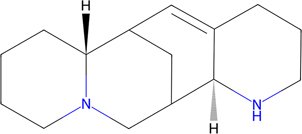	232.37	MA-104 cells PK-15 cells	*in vitro*	ASFV strain GZ201801	Aloperine specifically inhibits ASFV replication during both the mid-stage (syncytial infection) and late-stage (internalization) of the viral life cycle by inhibiting the prolactin receptor and activating the JAK2 signaling pathway.	[59]
Berbamine	C_37_H_40_N_2_O_6_	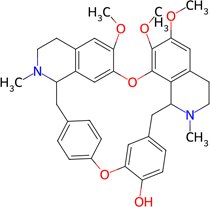	608.72	Vero cells Pams	*in vitro*	ASFV strain Armenia/07	Berbamine blocks ASFV entry (attachment/internalization), reduces viral protein synthesis (p30, p72), and suppresses pro‑inflammatory cytokines (TNF‑α, IL‑1β, IL‑6).	[60]
Berbamine hydrochloride	C_37_H_42_Cl_2_N_2_O_6_	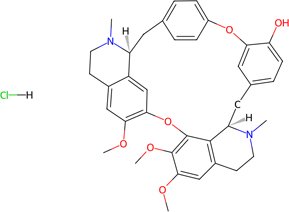	681.65	Pams 3D4/21 cells PK-15 cells	*in vitro*	ASFV strain GZ201801	Berbamine hydrochloride inhibits ASFV through dual mechanisms: blocking the early stage of viral invasion and interfering with subsequent viral replication.	[61]
Deoxycholic acid	C_24_H_40_O_4_	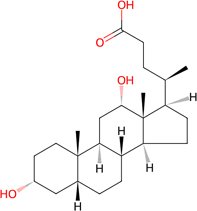	392.57	Pams	*in vitro*	ASFV strain GZ201801	Deoxycholic acid suppresses ASFV replication by interfering with viral protein I73R and transcription factor AP-1 interaction, thereby blocking AP-1 nuclear translocation and subsequently inhibiting MAPK pathway activation (p38, ERK, JNK phosphorylation).	[62]
Dihydromyricetin	C_15_H_12_O_8_	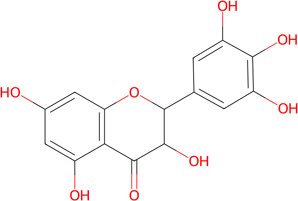	320.25	Pams	*in vitro*	ASFV strain GZ201801	Dihydromyricetin regulates TLR4/MyD88/MAPK/NF‑κB and ROS/NLRP3 to suppress ASFV‑induced cytokines (TNF‑α, IL‑1β, IL‑6, IL‑18) and inhibit pyroptosis (via GSDMD cleavage blockade).	[63]
Emodin	C_15_H_10_O_5_	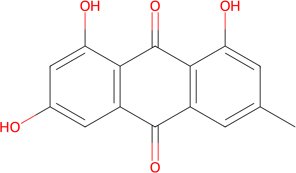	270.24	Pams	*in vitro*	ASFV strain GZ201801	Modin exerts its anti-ASFV activity by targeting Rab7, thereby disrupting the endocytic pathway and impairing viral attachment and internalization. It induces cholesterol accumulation and inhibits late endosome acidification, thereby blocking viral escape and suppressing ASFV replication.	[64]
Fangchinoline	C_37_H_40_N_2_O_6_	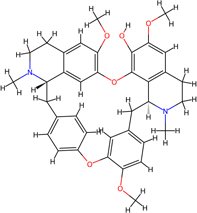	608.72	Pams	*in vitro*	ASFV strain GZ201801	Fangchinoline suppresses ASFV replication by inhibiting the AKT/mTOR/NF‑κB pathway: blocks AKT (Ser473) and mTOR (Ser2448) phosphorylation, downregulates p‑p65/p‑IκBα, and reduces pro‑inflammatory cytokines (IL‑1β, TNF‑α).	[65]
Genistein	C_15_H_10_O_5_	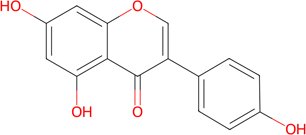	270.24	Alveolar cells Vero cells	*in vitro*	ASFV Ba71V strain ASFV strain Armenia/07	Genistein effectively inhibits ASFV replication by specifically targeting ASFV-topo II, competitively inhibiting its ATP-binding site, and inducing viral DNA double-strand breaks, thereby blocking viral DNA synthesis and late gene expression.	[66]
Genkwanin	C_16_H_12_O_5_	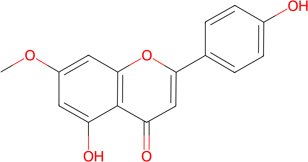	284.27	Vero cells	*in vitro*	ASFV strain Ba71V	Genkwanin targets two ASFV life cycle stages: inhibits viral entry (attachment/internalization) and suppresses egress by binding to the colchicine site of β‑tubulin to block microtubule polymerization, thereby reducing viral titers and suppressing viral DNA/protein synthesis.	[67]
Glycerol monolaurate	C_15_H_30_O_4_	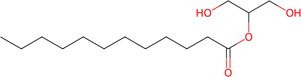	274.40	Pams	*in vitro*	ASFV strain Armenia/07	Glycerol monolaurate blocks ASFV infection by irreversibly disrupting the bilayer phospholipid structure of the viral envelope through the formation of micelles, leading to a significant reduction in viral titers.	[68]
Lupenone	C_30_H_48_O	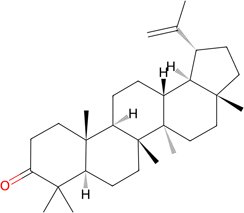	424.70	Vero cells	*in vitro*	ASFV strain Lisbon 60	Lupenone demonstrates antiviral activity against ASFV.	[69]
Luteolin	C_15_H_10_O_6_	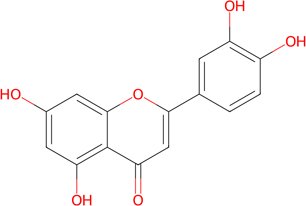	286.24	Pams	*in vitro*	ASFV strain GZ201801	Luteolin dose-dependently inhibits ASFV replication during the mid-to-late stage of infection by targeting the NF-κB/Signal transducer and activator of transcription 3 (STAT3)/ATF6 signaling network, effectively suppressing NF-κB phosphorylation, IL-6 secretion, STAT3 activation, and ATF6 processing.	[70]
Myricetin	C_15_H_10_O_8_	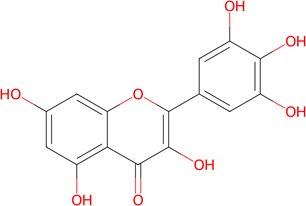	318.24	*E. Coli* BL21 (DE3) cells	*in vitro*	ASFV strainNP_042804.1	Myricetin and myricitrin inhibit ASFV particle maturation by specifically binding to the ASFV protease via their 3,4,5-trihydroxyphenyl group, thereby blocking the cleavage and processing of the viral polyproteins pp220 and pp62.	[71]
Myricitrin	C_21_H_20_O_12_	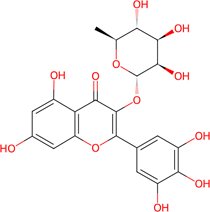	464.38					
Punicalin	C_34_H_22_O_22_	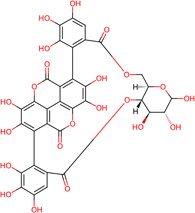	782.53	MA-104 cells PK-15 cells WSL-R4 cells Ipams	*in vitro*	ASFV strain GZ201801	Punicalin reduces ASFV-induced inflammation and suppresses viral replication by targeting the early stages of viral replication (attachment and internalization), directly inactivating virions, and modulating the NF-κB/STAT3/NLRP3 inflammasome signaling pathway.	[72]
Resveratrol	C_14_H_12_O_3_	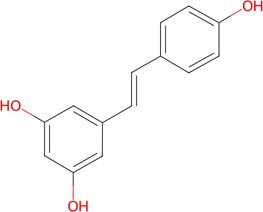	228.25	WSL cells Pams	*in vitro*	ASFV strain HLJ/2018	Resveratrol directly inhibits ASFV replication and alleviates oxidative damage by activating the Nrf2 signaling pathway, which disrupts Keap1-mediated degradation, promotes the expression of γ-GCS and SLC7A11 to increase GSH synthesis, and consequently facilitates ROS clearance.	[73]
Rhapontigenin	C_15_H_14_O_4_	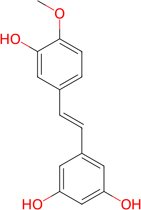	258.27	Pams	*in vitro*	ASFV strain GZ201801	Rhapontigenin significantly reduces ASFV proliferation by locking Rab7 to disrupt vesicular trafficking, thereby blocking viral entry. Concurrently, it elevates cholesterol levels within endosomal lipid rafts and inhibits endosomal acidification, ultimately restricting viral escape.	[64]
Rhein	C_15_H_8_O_6_	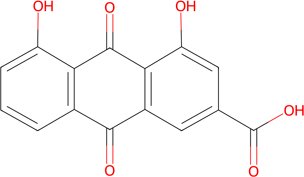	284.22	Pams	*in vitro*	ASFV strain GZ201801	Rhein induces mitochondrial ROS production, activates the caspase-9/3 cascade (upregulating Bax and downregulating Bcl-2), and triggers apoptosis in ASFV-infected macrophages, thereby inhibiting viral replication.	[74]
Tetrandrine	C_38_H_42_N_2_O_6_	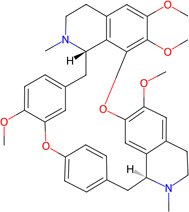	622.75	Pams MA-104 cells Vero cells MDBK cells IPEC-J2 cells	*in vitro*	ASFV strain anhuixcgq	Tetrandrine dose-dependently blocks ASFV entry into host cells via the macropinocytosis pathway by selectively inhibiting the PI3K/Akt signaling axis.	[75]
Theaflavin	C_29_H_24_O_12_	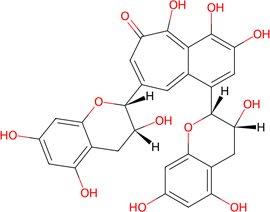	564.49	Pams	*in vitro*	ASFV strain GZ201801	Theaflavin activates the AMPK signaling pathway, leading to significant downregulation of lipid synthesis-related genes. This process reduces the intracellular accumulation of total cholesterol and triglycerides, disrupting the lipid metabolism environment that is essential for ASFV replication and thereby suppressing viral proliferation.	[76]
Thonningianin A	C_42_H_34_O_21_	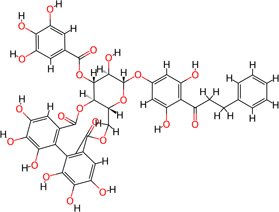	874.71	Pams	*in vitro*	ASFV strain CN/GS/2018	Thonningianin A binds to the DNA-binding domain of pA104R, forming a strong hydrogen bond with H100 and occupying critical DNA-binding residues K92, R94, and K97, resulting in significant inhibition of ASFV replication.	[77]
Toosendanin	C_30_H_38_O_11_	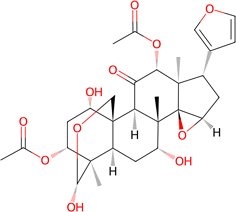	574.62	Pams	*in vitro*	ASFV strain HLJ/2018	Toosendanin upregulates IRF1 expression to activate the interferon pathway (IFN-α/β/γ), thereby inhibiting both the internalization and replication of ASFV and reducing viral protein synthesis, genomic load, and viral titers.	[78]

Note: The chemical structures mentioned in the table are from https://www.chemspider.com/.

#### Effects of natural compounds against CSFV

Natural compounds with antiviral activity against CSFV can be divided into mixtures (*Astragalus* polysaccharides, *Paulownia tomentosa* flower polysaccharides, *Cordyceps militaris* (*C. militaris*) ethanol extract, and traditional Chinese medicine prescriptions such as Xiao Jian Zhong prescriptions and Jingsananli-sepsis) and monomeric compounds (ginsenosides, curcumin and saponins).

##### Attachment and entry

*Astragalus* polysaccharides block E2 protein-mediated attachment and internalization, thereby preventing viral entry [79].

##### Replication

Following viral entry into the cell, ginsenosides Rb2/Rb3 specifically target the viral internal ribosome entry site to inhibit translation initiation and block viral protein synthesis [80].

##### Host-directed therapy

*Astragalus* polysaccharides suppress the overexpression of clusters of differentiation 28 (CD28)/cytotoxic T lymphocyte-associated antigen 4, modulate the interleukin-2 (IL-2)/transforming growth factor-β (TGF-β) balance, and activate the toll-like receptor 4 (TLR4)/NF-κB pathway, thereby maintaining immune homeostasis and exerting antioxidant effects while simultaneously acting as an adjuvant to increase antibody levels [81–83]. Additionally, polysaccharides in *P. tomentosa* flower increase NK cell cytotoxicity and the T helper 1 cell/T helper 2 cell (Th1/Th2) immune response, leading to elevated antibody levels [84]. Xiao Jian Zhong prescriptions, Jingsananli-sepsis, and *C. militaris* ethanol extract, when used as feed additives, can increase antibody levels in serum, thereby enhancing immunity [85,86]. Traditional Chinese medicines have complex compositions with undefined active ingredients, and their adjuvant activity may arise from multicomponent synergy; however, their long-term safety remains unknown.

With respect to monomeric compounds, saponins derived from a water extract of Quillaja can also serve as vaccine adjuvants to increase serum antibody levels [87]. Curcumin (<15 μM) inhibits fatty acid synthase (FASN) to reduce lipid droplet accumulation and downregulates the expression of activating transcription factor 6 (ATF6) as well as its downstream molecules C/EBP-homologous protein (CHOP) and glucose-regulated protein (GRP78), thereby disrupting virus-dependent lipid metabolism. Moreover, curcumin activates the interferon regulatory factor 3 (IRF3) / interferon regulatory factor 7 (IRF7)-signal transducer and activator of transcription 1 (STAT1)-interferon-stimulated gene 15 (ISG15) pathway to upregulate antiviral protein expression, establishing the first line of host defense [88]. Curcumin has poor oral absorption and rapid metabolism. Despite an effective concentration of <15 μM *in vitro*, whether this concentration can be achieved and maintained for a sufficient duration *in vivo* remains inadequately addressed, which severely limits its feasibility for clinical translation.

#### Effects of natural compounds against FMDV

This section presents an overview of natural compounds with demonstrated antiviral activity against FMDV, which can be divided into mixtures and monomeric compounds. Mixtures (*Moringa oleifera* (*M. oleifera*) and *Azadirachta indica* (*A. indica*)) offer advantages such as multitarget synergy, lower cost, and potentially reduced toxicity; however, their mechanisms are unclear and the batch-to-batch consistency is poor. In contrast, the mechanisms of monomeric compounds (cephaeline, emetine, homoharringtonine, andrographolide, luteolin, isoginkgetin, brusatol, nigericin, mizoribine, apigenin, ovine interferon tau 4, and quercetin) are clear; in addition, they are highly pure and suitable for drug development. However, these compounds can induce viral resistance, often show low bioavailability, and may exhibit high toxicity when isolated from the natural matrix.

##### Direct inactivation

Prior to FMDV invasion, cephaeline and emetine directly inactivate free virions [89]. These alkaloids have been traditionally used as emetics. Their emetic dose may overlap with the effective antiviral dose, and the underdeveloped vomiting reflexes in ruminants may lead to secondary injuries such as ruminal tympany [90].

##### Attachment and entry

The ethanol extract of *Spirulina platensis* inhibits viral entry [91].

##### Replication

Following viral entry into cell, multiple monomers inhibit viral replication through different mechanisms. Homoharringtonine rapidly blocks early viral translation and replication [92]. Homoharringtonine is an approved anticancer drug with known hematological toxicity [93]. Therefore, its use in livestock requires careful dose adjustment and residue monitoring. Andrographolide and its derivative deoxyandrographolide bind to the active site (His46/Cys163) of the 3C protease (3Cpro), whereas luteolin and isoginkgetin inhibit 3Cpro-mediated polyprotein processing, thereby disrupting viral proteolytic maturation [94,95]. During the viral RNA replication stage, brusatol and bruceine D target the catalytic site of 3D polymerase (3Dpol), whereas nigericin, cephaeline, and emetine inhibit the activity of 3Dpol, blocking viral RNA synthesis [89,96,97]. Mizoribine further suppresses early replication by inhibiting inosine-5’-monophosphate dehydrogenase (IMPDH)-dependent purine synthesis [98]. Additionally, its *in vivo* protective effect was preliminarily validated in a suckling mouse model. At the translational level, apigenin selectively inhibits FMDV IRES-mediated protein translation without affecting RNA transcription [99].

##### Host-directed therapy

Ovine interferon tau 4 and quercetin activate the Janus kinase (JAK)-signal transducer and activator of transcription (STAT) and type I interferon-alpha/beta (IFN-α/β) pathways, respectively, upregulating the expression of antiviral proteins such as ISG15 and MX dynamin-like GTPase (MX1), which collaboratively facilitate viral clearance [100,101].

Among the tested mixtures, *M. oleifera* and *A. indica* extracts exhibit potent anti-FMDV activity [102]. The precise mechanism of action of this mixture, including the identification of the specific stage it acts on (direct inactivation, attachment, entry, replication, release, or host-directed therapy), requires further investigation.

#### Effects of natural compounds against JEV

This section summarizes relevant studies on natural compounds with anti-JEV activity. However, most of the compounds discussed here are crude extracts or mixtures, such as crude plant extracts and marine algal extracts. Plant extracts that have demonstrated anti-JEV activity include *Rhodiola crenulata* (*R. crenulata*) extract, pokeweed extract, belladonna formulation, and *Astragali Radix* (*A. Radix*) extract. Marine algal extracts with confirmed anti-JEV activity include extracts from *Gracilaria sp*. and *Monostroma nitidum* (*M. nitidum*), sulfated polysaccharide extracts from *Brasenia schreberi*, and the compounds carrageenan and griffithsin. These compounds lack well-defined monomeric components. This absence of chemical characterization severely limits their potential for clinical translation.

##### Direct inactivation

Among marine alga-derived substances, sulfated polysaccharide extracts from *Ulva lactuca*, which are characterized by a low degree of polymerization, directly bind to JEV particles. This binding effectively inhibited JEV infection and increased survival rates in JEV-infected mice [103].

##### Attachment and entry

Prior to JEV invasion, *R. crenulata* extract interferes with virus–receptor binding and cellular entry [104]. Carrageenan inhibits JEV infection at the attachment and cellular entry stages [105]. Griffithsin, a natural lectin identified in the red alga *Griffithsia* sp., prevents viral entry into host cells by binding to viral glycans. Specifically, griffithsin interacts with glycosylated JEV proteins, thereby inhibiting JEV infection [106,107].

##### Replication

Following viral entry, multiple agents interfere with JEV replication. Belladonna formulation directly suppresses NS3 protein expression, reducing overall viral replication [108]. The tropane alkaloids present in belladonna have well‑defined anticholinergic toxicity, a property that necessitates strict control during their use in livestock [109].

##### Host-directed therapy

Various substances exert anti-JEV effects by modulating host immunity, inflammation, or cell survival. Belladonna formulation reduces apoptosis, inhibits microglial activation, diminishes neuronal cell death, and interferes with tumor necrosis factor (TNF)-induced NF-κB signal transduction [108,110]. The crude extract of *A. Radix* administered via intraperitoneal injection effectively inhibited JEV infection throughout the infection cycle and increased the survival rate of mice [111]. However, intraperitoneal injection is not a practical route of administration for livestock. Sulfated saccharides extracted from *Gracilaria* sp. and *M. nitidum* exhibit anti-JEV effects, although their precise mechanisms remain unclear, and their active components are undefined [112].

Among the monomers, pokeweed antiviral protein (isolated from pokeweed) stands out as a unique proteinaceous agent. It directly degrades viral genetic material by catalyzing the depurination of viral RNA, thereby decreasing the capacity of JEV to replicate [113]. However, pokeweed antiviral protein is a potent toxin that also depurates host ribosomes, leading to severe cytotoxicity [114]. Its protein nature and strong toxicity distinguish it from conventional small-molecule compounds and limit its direct application in livestock without rigorous safety evaluation.

In contrast, compounds with clearly identified monomeric structures allow for clear structural characterization, facile elucidation of their mechanisms of action, and greater reproducibility and translational value. Therefore, they are summarized separately in Table 3. In total, 32 monomeric compounds with confirmed anti-JEV activity and their corresponding mechanisms of action are detailed in Table 3.

**Table 3.** Natural active ingredients with anti-JEV activity.

Name	Molecular Formula	Molecular Structure	Molecular Weight (g/mol)	Experimental Model (Cell/Animal)	Tested Range	Virus Strain	Mechanism of Action	Cite
Aloe-emodin	C_15_H_10_O_5_	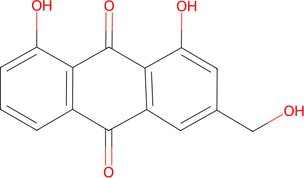	270.24	Vero cells BHK 21 cells	*in vitro*	JEV strain T1P1	Aloe-emodin exerts indirect antiviral effects by directly inactivating virions and enhancing the host’s innate immune response through activation of the gamma-interferon activation sequence (GAS)-driven promoter.	[115]
Andrographolide	C_20_H_30_O_5_	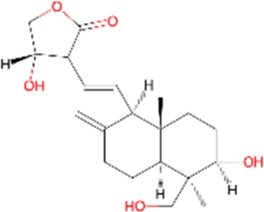	350.45	/	*in vitro*	/	Andrographolide inhibits the NS3 protease of JEV in a concentration-dependent manner.	[116]
Arctigenin	C_21_H_24_O_6_	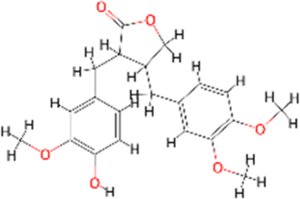	372.41	BALB/c mice Neuro-2a cells	*in vivo* *in vitro*	JEV strain GP78	Arctigenin exerts direct antiviral activity by suppressing JEV replication and reducing viral load while concurrently providing neuroprotection through the modulation of oxidative stress and inflammatory responses, significantly reducing neuronal cell death and gliosis.	[117]
Artemisinin	C_15_H_22_O_5_	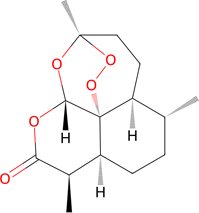	282.33	BHK cells C57BL6J mice	*in vivo* *in vitro*	JEV strain P3	Artemisinin and its derivative artesunate significantly reduce mortality in JEV-infected mice and alleviate JEV-induced brain damage. *In vitro*, artemisinin markedly inhibits JEV replication and reduces virion production.	[118]
Artesunate	C_19_H_28_O_8_	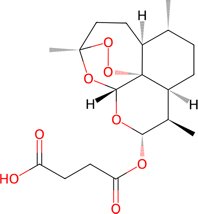	384.42					
Baicalein	C_21_H_18_O_12_	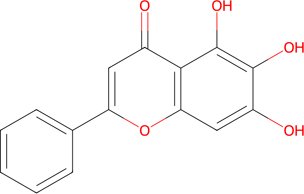	446.36	Vero cells	*in vitro*	JEV strainHE861351	Baicalein exhibits potent anti-JEV activity by blocking viral attachment, directly inactivating virions, and inhibiting viral replication.	[119]
Berbamine	C_37_H_40_N_2_O_6_	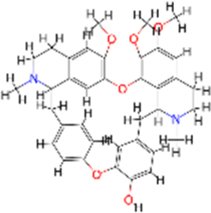	608.72	Vero cells BHK 21 cells, 4T1 cells	*in vitro*	JEV strain SA14-14–2	Berbamine inhibits JEV infection by interfering with TRPMLs-mediated endosomal–lysosomal trafficking of the low-density lipoprotein receptor	[120]
Cepharanthine	C_37_H_38_N_2_O_6_	/	606.70	BHK 21 cells Vero cells	*in vitro*	JEV strain SA14-14–2	Cepharanthine inhibits viral RNA replication by competitively binding to the active site of the RdRp with GTP.	[121]
Chondroitin sulfate-E	(C_14_H_21_NO_16_S_2_)_n_	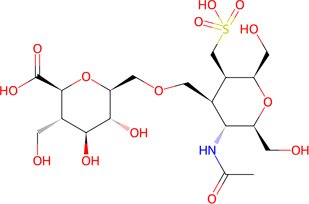	694.5n	BHK 21 cells, Vero cells,Neuro-2a cells	*in vivo* *in vitro*	JEV strainNakayama	The effect of chondroitin sulfate-E on JEV infection is cell type-dependent, exhibiting paradoxical roles: it inhibits viral infection in nonneuronal cells while promoting infection in neuronal cells.	[122]
Chrysophanol	C_15_H_10_O_4_	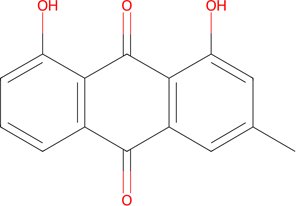	254.24	Vero cells BHK 21 cells	*in vitro*	JEV strain T1P1	Chrysophanol directly inactivates virions and enhances innate immunity by activating the GAS promoter.	[115]
Corosolic acid	C_30_H_48_O_4_	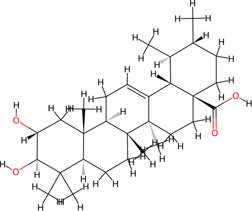	472.71	BHK cells Vero cells Neuro-2a cells PK-15 cells HEK-293Tcells Three-week-old C57BL/6 mice	*in vivo* *in vitro*	JEV strain NJ2008 JEV strain SA14-14–2	Corosolic acid significantly inhibits JEV replication by activating the AMPK signaling pathway and suppressing lipid synthesis, demonstrating protective effects against JEV infection *in vivo*.	[123]
Curcumin	C_21_H_20_O_6_	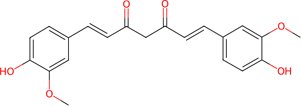	368.38	BHK 21 cells, Neuro-2a cells	*in vitro*	JEV strain GP05	Curcumin exerts direct antiviral activity by inhibiting JEV NS5 protein expression and modulating host pathways (IL6/AKT1/TNF-α/PTGS2). It concurrently inhibits apoptosis by reducing oxidative stress, maintaining cell membrane integrity, and downregulating the phosphorylation levels of p38 MAPK, ERK1/2, and JNK. Furthermore, it reduces infectious virion production via the ubiquitin-proteasome system	[124,125]
Daidzin	C_21_H_20_O_9_	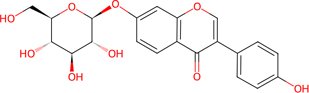	416.38	BHK 21 cells	*in vitro*	JEV strain SA14-14–2	Daidzin directly inhibits JEV replication by binding to the viral frameshift site RNA.	[126]
Digoxin	C_41_H_64_O_14_	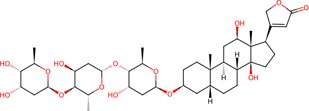	780.95	BHK 21 cells Vero cells Huh-7 cells BALB/c mice	*in vivo* *in vitro*	JEV strain AT31 JEV strain SA14	Digoxin blocks JEV infection at the replication stage by inhibiting Na^+^/K^+^-ATPase.	[127]
Epigallocatechin-3-gallate	C_22_H_18_O_11_	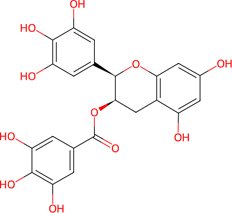	458.37	BHK 21 cells, TE671 cells	*in vitro*	JEV strains T1P1,JEV strains PK1	Epigallocatechin-3-gallate significantly inhibits JEV attachment and entry.	[128]
Genistein	C_15_H_10_O_5_	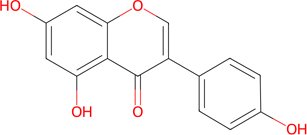	270.24	Neuron/glia cultures were prepared from cerebral cortices of 1-day-old Sprague–Dawley rats	*in vitro*	/	Genistein suppresses the production of JEV-induced inflammatory factors and the activation of NF-κB, alleviating the neurotoxicity associated with JEV infection. However, it failed to affect JEV replication.	[129]
Hyoscyamine	C_17_H_23_NO_3_	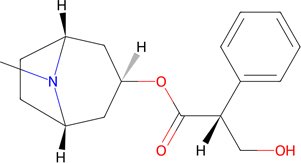	289.37	Vero cells Embryonated eggs	*in vivo* *in vitro*	JEV strain SH1	Hyoscyamine inhibits viral replication and elicits an anti-inflammatory response by stably binding the NS5 protein, modulating TLRs (downregulating TLR4 and upregulating TLR3, TLR7, TLR8), and regulating the JAK-STAT pathway.	[130]
Indigo	C_16_H_10_N_2_O_2_	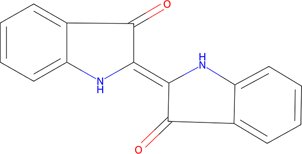	262.26	Vero cells BHK-21 cells HL-CZ cells	*in vitro* *in vivo*	JEV strain T1P1	Indigo and indirubin inhibit JEV by blocking viral attachment and directly inactivating viral particles. Indirubin exhibits broad-spectrum antiviral activity and demonstrates superior efficacy compared to other components in both *in vitro* and *in vivo* experiments.	[131]
Indirubin	C_16_H_10_N_2_O_2_	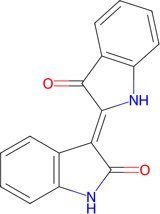	262.26					
Isobavachalcone	C_20_H_20_O_4_	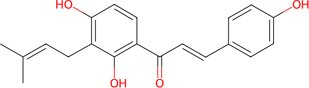	324.38	BHK cells Vero cells Neuro-2a cells PK-15 cells HEK-293Tcells Three-week-old C57BL/6 mice	*in vivo* *in vitro*	JEV strain NJ2008 JEV strain SA14-14–2	Isobavachalcone significantly inhibits JEV replication by activating the AMPK signaling pathway and suppressing lipid synthesis, showing protective effects *in vivo* against JEV infection.	[123]
Luteolin	C_15_H_10_O_6_	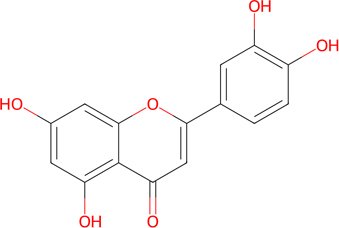	286.24	A549 cells BHK 21 cells	*in vitro*	JEV strain SX09S01	Luteolin exhibits virucidal activity against extracellular JEV particles.	[132]
Melatonin	C_13_H_16_N_2_O_2_	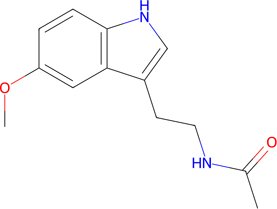	232.28	SH-SY5Y cells Vero cells SK-N-SH cells	*in vitro*	JEV strain Beijing	Melatonin directly targets viral NS3/NS5 domains to inhibit replication, and modulates calcineurin to balance autophagy, thereby alleviating neuroinflammation and virus-induced neuronal apoptosis, conferring neuroprotection.	[133,134]
Mycophenolic acid	C_17_H_20_O_6_	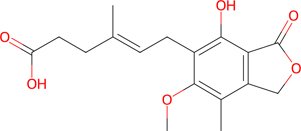	320.34	PS cells Swiss albino mice	*in vivo* *in vitro*	JEV strain P20778	Mycophenolic acid specifically interferes with JEV RNA replication (primarily at late stages) by inhibiting IMPDH to block nucleotide synthesis and shows significant antiviral protection in mouse models.	[135]
Ouabain	C_29_H_44_O_12_·8 H_2_O	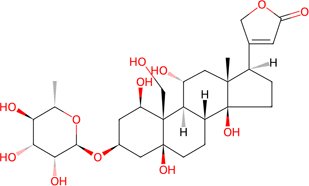	728.77-	BHK 21 cells Vero cells Huh-7 cells BALB/c mice	*in vivo* *in vitro*	JEV strain AT31 JEV strain SA14	Ouabain blocks JEV replication by targeting Na^+^/K^+^-ATPase, reduces viral load, alleviates pathological damage, and significantly improves survival rates in infected mice.	[127]
Pancratistatin	C_16_H_17_NO_8_	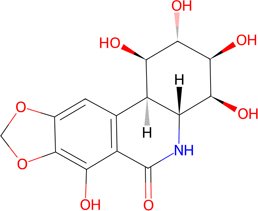	325.27	MT-2 cells CEM cells Vero cells	*in vivo* *in vitro*	JEV strain Nakayama	Pancratistatin inhibit JEV replication and increase survival rates in infected mice.	[136]
Quercetin	C_15_H_10_O_7_	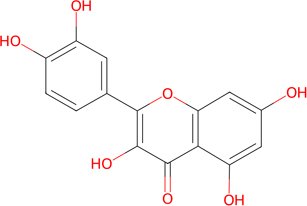	302.24	Vero cells	*in vitro*	JEV strain HE861351	Quercetin exhibits only limited inhibitory effects post-viral attachment.	[119]
Rosmarinic acid	C_18_H_16_O_8_	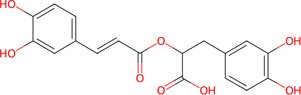	360.31	5-week BALB/c mice Neuro-2a cells BV-2 cells	*in vivo* *in vitro*	JEV strain GP78,JEV strain Nakayama JEV strain HW1	Rosmarinic acid increases survival of JEV-infected mice by reducing viral load, suppressing inflammatory cytokines (IL‑12, TNF‑α, IFN‑γ, MCP‑1, IL‑6), and decreasing microglial activation. *In vitro*, it inhibits JEV replication and alleviates inflammation by downregulating RIG‑I/p62 to block NF‑κB.	[137,138]
Rutin	C_27_H_30_O_16_	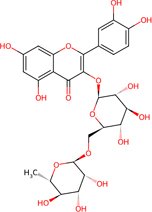	610.521	Vero cells C57BL/6 WT mice	*in vivo* *in vitro*	JEV strain P20778	Rutin shows virucidal activity, blocks viral entry (E protein Domain III), inhibits NS3 protease, and reduces neuroinflammation (microglial activation, NLRP3, cytokines, ROS), thereby improving survival and encephalitis in JEV-infected mice.	[139]
Scopolamine	C_17_H_21_NO_4_	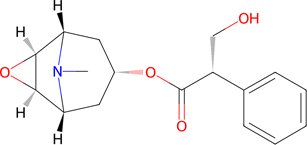	303.35	Vero cells 1-day-old embryonated chick eggs (Gallus gallus)	*in vivo* *in vitro*	JEV strain SH1	Scopolamine synergistically inhibits viral replication by directly binding the JEV NS5 protein and through dual immunomodulation (activating TLR3/7/8 and IFN-α/β pathways, enhancing IL-4/10 expression), thereby strengthening the antiviral immune response.	[140]
Sodium salicylate	C_7_H_5_nao_3_	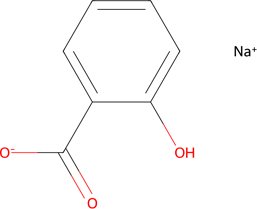	160.14	BHK cells N18 cells	*in vitro*	JEV strain NT113	Sodium salicylate significantly inhibits JEV replication and cytotoxicity by activating the MAPK signaling pathway, an effect independent of COX activity inhibition.	[141]
(-)-tetrahydropalmatine	C_21_H_25_NO_4_	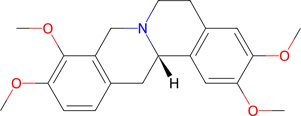	355.43	4–5 weeks BALB/c mice Neuro-2a cells	*in vivo*	JEV strain GP78	(-)-Tetrahydropalmatine demonstrates significant antiviral and neuroprotective effects by reducing viral load, inhibiting microglial activation, and decreasing inflammatory mediators and oxidative stress.	[142]
2-methylnaphtho[2,3-b]furan-4,9-dione	C_13_H_8_O_3_	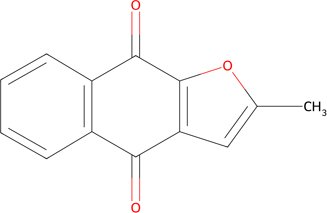	212.20	BHK cells Vero cells	*in vitro*	JEV strain jagar-01	2-Methylnaphtho[2,3-b]furan-4,9-dione inhibits JEV replication by suppressing the synthesis of viral RNA and proteins.	[143]
7-Deoxypancratistatin	C_16_H_17_NO_7_	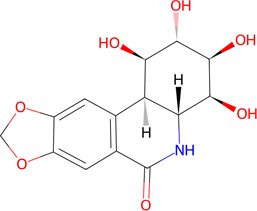	309.274	MT-2 cells CEM cells Vero cells	*in vivo* *in vitro*	JEV strain Nakayama	7-Deoxypancratistatin inhibit JEV replication and increase survival rates in infected mice.	[136]

Note: The chemical structures mentioned in the table are from https://www.chemspider.com/.

#### Effects of natural compounds against PCV2

This section presents a review of natural compounds that exhibit anti‑PCV2 activity, primarily crude extracts or fractions from natural sources. However, most of these compounds lack well‑defined monomeric components. This absence of chemical characterization severely limits their pharmacological reproducibility, batch‑to‑batch consistency, and potential for clinical translation, as the actual active ingredients remain unknown. The compounds discussed include *Panax notoginseng* (*P. notoginseng*) saponins, total flavonoids of *Spatholobus suberectus* (*S. suberectus*) Dunn, *Polygonum hydropiper* L (*P*. *hydropiper* L.). extract, fermentation products of *Ganoderma lucidum* (*G. lucidum*), saikosaponins A/D, anemoside B4, various polysaccharides (from *Sargassum*, *Atractylodes macrocephala* (*A. macrocephala*), *Platycodon grandiflorus* (*P. grandiflorus*), *Astragalus*, and *Sophora subprostrate*), and liposome‑based vaccine adjuvants.

##### Attachment and entry

Among these mixtures, the ethyl acetate fraction of flavonoids from *P. hydropiper* L. rapidly inhibits NF-κB nuclear factor kappa-B subunit p65 (p65) nuclear translocation and inhibitor of nuclear factor kappa B alpha (IκBα) degradation, thereby blocking NF-κB pathway activation. This fraction also decreases Ak strain-transforming (Akt) phosphorylation to inhibit the phosphatidylinositol 3-kinase/protein kinase B (PI3K/Akt) signaling cascade, attenuating the inflammatory microenvironment required for early viral replication [144].

##### Host-directed therapy

This section summarizes natural mixtures that exert anti-PCV2 effects by modulating the balance of histone acetylation and the oxidative stress pathway. *P. notoginseng* saponins reverse the PCV2‑induced imbalance of histone acetylation (by inhibiting histone acetyltransferases or activating deacetylases) and directly regulate oxidative stress molecules as well as related enzyme activities, thereby reducing oxidative damage [145,146]. Moreover, total flavonoids of *S. suberectus* Dunn regulate the enzymes associated with oxidative stress, alleviating PCV2-induced cellular injury [147,148]. Various polysaccharides (from *Sargassum*, *P. grandiflorus* and *Sophora subprostrate*) can regulate the histone acetyltransferase (HAT)/histone deacetylase (HDAC) balance and inhibit the NF-κB pathway, thereby suppressing the transcription of inflammatory genes [149,150]. *P. grandiflorus* polysaccharide additionally inhibits MAPK signaling [151]. *Sophora subprostrate* polysaccharide exerts its effects by regulating the lncRNA/miRNA/mRNA network and inhibiting histone acetylation. This polysaccharide inhibits the NF-κB/MAPK pathway, increases the glutathione (GSH)/glutathione disulfide ratio, scavenges hydroxyl radicals, and decreases reactive oxygen species (ROS) and nitric oxide levels as well as myeloperoxidase and inducible nitric oxide synthase activity, thereby protecting mitochondrial function. Furthermore, this polysaccharide persistently suppresses inflammation and viral replication at the posttranscriptional level via the miR-328-y/ tumor necrosis factor‑alpha (TNF‑α) axis and the NF-κB/TNF and MAPK/c-Jun networks [152–158].

In the autophagic process, *A. macrocephala* polysaccharide inhibits the PTEN-induced putative kinase 1/Parkin pathway-mediated mitophagy, thereby disrupting the ability of PCV2 to exploit autophagy to promote its replication [159].

This section summarizes natural mixtures that exert anti-PCV2 effects by regulating the expression of inflammatory factors and enhancing immune responses. The n-butanol extract of *P. hydropiper* L. downregulates the expression of proinflammatory factors while upregulating IL-10 expression. This process significantly attenuates the inflammatory response during the viral replication and dissemination phase, protecting against tissue damage in animals [160]. Furthermore, when used as a feed additive at 50 mg/kg, fermented products of *G. lucidum* increase the expression of interferon‑gamma (IFN‑γ) and TNF-α, accelerate viral clearance, maintain the structural integrity of lymphoid tissues, and provide structural support for the host’s antiviral defense [161]. *Astragalus* polysaccharides inhibit the activation of the NF-κB pathway [162] and synergistically suppress endoplasmic reticulum stress and oxidative stress, thereby blocking the viral replication cycle at multiple targets [163].

Natural compounds serve as vaccine adjuvants to establish long-lasting protection by modulating immune responses. Vaccine adjuvants such as salidroside liposomes, *Rehmannia glutinosa* polysaccharide liposomes, *Epimedium* polysaccharide-propolis flavone liposomes, and Chinese yam polysaccharides can further increase antibody titers, balance the T helper type 1 (Th1)/ T helper type 2 (Th2) response, activate macrophages and dendritic cells, increase antigen delivery and T-cell activation, and ultimately establish a long-lasting immune barrier [164–167]. Although these substances are advantageous for enhancing immune responses as vaccine adjuvants, when used alone as antiviral drugs, their direct antiviral activity, low bioavailability, safety, and other aspects still require further investigation.

The various polysaccharides mentioned above have shown potential anti-PCV2 activity through multiple regulatory mechanisms. However, many polysaccharides have a high molecular weight, resulting in their poor absorption after oral administration and easy metabolic inactivation by the immune system, which reduces their efficacy. Moreover, studies on their long‑term toxicity, tissue residue, and metabolism are insufficient. Whether their prolonged use in food‑producing animals leads to accumulation in the body and subsequently poses a risk to human health through the food chain remains unknown.

In contrast, compounds with clearly identified monomeric structures, such as the 12 compounds listed in Table 4, allow for unambiguous chemical characterization, facile elucidation of their mechanisms of action, and greater reproducibility and translational value. Therefore, they are summarized separately in Table 4, which details these eight monomeric compounds and their corresponding anti-PCV2 mechanisms.

**Table 4.** Natural active ingredients with anti-PCV2 activity.

Name	Molecular Formula	Molecular Structure	Molecular Weight (g/mol)	Experimental Model (Cell/Animal)	Tested Range	Virus Strain	Mechanism of Action	Cite
Anemoside B4	C_59_H_96_O_26_	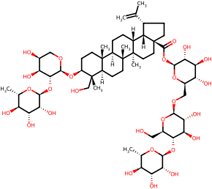	1221.39	Porcine iliac artery endothelial cells BALB/c mice	*in vivo* *in vitro*	PCV2 strain AH	Anemoside B4 inhibits IL-10, thereby upregulating IgG, restoring white blood cells, and alleviating pathological damage during PCV2 infection, thus relieving immunosuppression and suppressing the pathogenic effects of the virus.	[168,169]
Arctigenin	C_21_H_24_O_6_	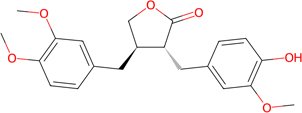	372.41	PK-15 cells BALB/c female mice	*in vivo* *in vitro*	PCV2 strain WH	Arctigenin significantly reduces viral load in the tissues of infected mice by directly inhibiting viral replication. It also alleviates PCV2-induced inflammatory responses by inhibiting IκBα phosphorylation and p65 nuclear translocation, thereby blocking the activation of the NF-κB pathway.	[170,171]
Curcumin	C_21_H_20_O_6_	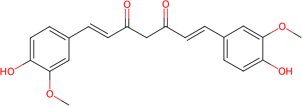	363.38	PK-15 cells	*in vitro*	PCV2 strain SH	Curcumin exerts anti-PCV2 effects by inhibiting the expression of the PCV2 capsid protein, modulating apoptosis-related proteins (cleaved caspase-3, Bax), and reducing ROS levels, thereby effectively blocking the virus-induced mitochondrial apoptosis pathway.	[172]
Epigallocatechin gallate	C_22_H_18_O_11_	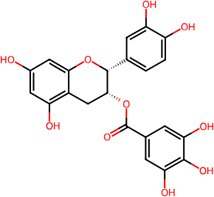	458.37	PK-15 cells	*in vitro*	PCV2 strain HZ0201	Epigallocatechin gallate exerts its antiviral effect against PCV2 by binding directly to the viral capsid protein, thereby competitively blocking its attachment to the host cell receptor heparan sulfate (HS) through key interactions with capsid residues ARG51, ASP70, ARG73, and ASP78.	[173]
Matrine	C_15_H_24_N_2_O	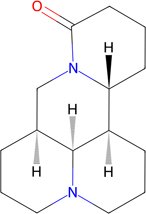	248.36	PK-15 cells PAM cells Kunming mice	*in vivo* *in vitro*	/	Matrine inhibits PCV2 replication by modulating the STAT3-Beclin1 signaling axis. In a PRRSV/PCV2 coinfection model, it significantly reduces hepatic viral load and alleviates interstitial pneumonia. Concurrently, it maintains intestinal epithelial barrier function and reduces inflammation by increasing the abundance of *L. acidophilus*, demonstrating multitarget antiviral activity.	[174–176]
Osthole	C_15_H_16_O_3_	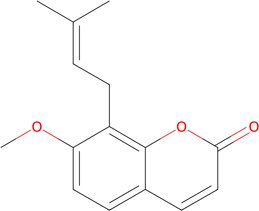	244.29	PK-15 cells Kunming mice	*in vivo* *in vitro*	PCV2 strain SH	The combination of matrine and osthole synergistically inhibits PCV2 replication by reducing Cap gene and protein expression. It exhibits a dual antiviral mechanism by downregulating the expression of GRP78, p-PERK, p-eIF2α, ATF4, and CHOP, inhibiting the pro-apoptotic proteins Bax and cleaved caspase-3, upregulating the anti-apoptotic protein Bcl-2, and modulating GRP78/PERK pathway-dependent apoptosis.	[177,178]
Paeoniflorin	C_23_H_28_O_11_	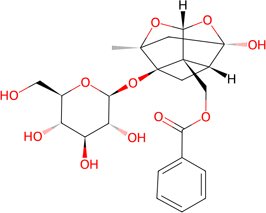	480.46	PK-15 cells	*in vitro*	PCV strain 2d	Paeoniflorin promotes AKT/mTOR phosphorylation, downregulates LC3‑II, and upregulates p62 to inhibit PCV2‑induced autophagy and reduce PCV2 replication.	[179]
Quercetin	C_15_H_10_O_7_	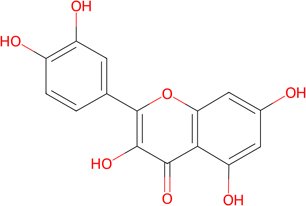	302.24	3D4/2 cells PK-15 cell	*in vitro*	The porcine circovirus II (PCV2) strain	Quercetin activates Nrf2/HO-1 for antioxidant, reduces histone acetylation, blocks NF-κB, and reduces p-p38/p-AKT to lower pro-inflammatory mediators and raise IL-10, linking Nrf2-HO-1 with PI3K/AKT.	[180]
Saikosaponin A	C_42_H_68_O_13_	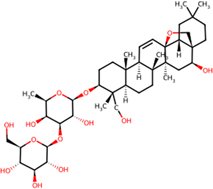	780.99	Porcine iliac artery endothelial cells BALB/c mice	*in vivo* *in vitro*	PCV2 strain AH	Saikosaponins A/D exert antiviral effects by enhancing Th1-type immune responses. In PCV2-infected cell and mouse models, they upregulate IL-2, IFN-γ, and serum IgG levels, restore white blood cell counts, and alleviate pathological damage such as fever, weight loss, and organ swelling. These results indicate that SSA and SSD effectively alleviate PCV2-induced immunosuppression and enhance host antiviral defense by promoting Th1-type cytokines and immunoglobulins.	[168,169]
Saikosaponin D	C_42_H_68_O_13_	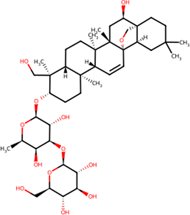	780.99					
Sanguinarine	C_20_H_14_NO_4_	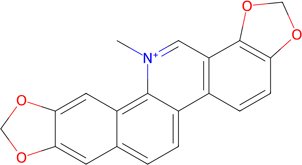	332.29	PK-15 cells	*in vitro*	PCV2 strain SH	Sanguinarine upregulates IFIH1 expression, promotes the phosphorylation of STAT1 and IRF3, and activates MAVS signaling, ultimately enhancing IFITM1 expression to inhibit viral replication. It also significantly inhibits the expression of p38α, JNK, and p-JNK proteins in PCV2-infected cells, blocking virus-induced apoptosis.	[181]
Taurine	C_2_H_7_NO_3_S	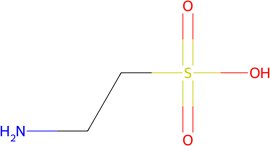	125.15	PK-15 cells	*in vitro*	The PCV2 strain(PCV2NJ2002)	Taurine inhibits viral replication through a molecular mechanism involving an antioxidant–autophagy regulatory axis: it reduces OTA-induced oxidative stress and ROS accumulation, and it modulates the AMPK/mTOR signaling axis to suppress autophagy by reducing LC3-II and ATG5 expression.	[182]

Note: The chemical structures mentioned in the table are from https://www.chemspider.com/.

#### Effects of natural compounds against PPV

Due to the limited number of studies on antiviral drugs against PPV, only four relevant articles are included for discussion in this subsection. Natural compounds with demonstrated antiviral activity against PPV include trimethylamine N-oxide, propolis flavone, and the monomeric compound ferulic acid.

##### Direct inactivation

Trimethylamine N-oxide (TMAO) is a naturally occurring osmolyte that is widely present in marine fish, mammals, and certain plants and microorganisms. Prior to PPV invasion, TMAO prevents the assembly of viral capsid proteins into infectious virions, thereby directly reducing PPV infectivity [183].

##### Replication

At this stage, both mixtures and monomeric compounds play a role. In terms of mixtures, propolis flavone inhibits PPV replication [184]. In terms of monomeric compounds, ferulic acid targets the viral NS1 protein, inhibits the TLR4/NF-κB pathway and ROS accumulation to block inflammasome activation, and suppresses PPV-activated p38 MAPK and c-Jun N-terminal kinase (JNK) signaling to prevent subsequent apoptosis [185].

##### Host-directed therapy

Propolis flavone enhances cellular immunity by upregulating the expression of IL-2, interleukin‑6 (IL‑6), and IFN-γ. When used as an adjuvant, propolis flavone activates both Th1 and Th2 immune responses, resulting in increased levels of the antibody subclasses IgM and IgG [186].

#### Effects of natural compounds against PRRSV

This section presents a review of relevant research on natural compounds with anti-PRRSV activity, including plant extracts, marine-derived natural ingredients, and traditional Chinese medicine formulations. Most of these compounds are crude extracts or complex mixtures without well‑defined monomeric components, and a lack of chemical characterization greatly hinders their clinical translation. Plant extracts (*Cynodon dactylon* (*C. dactylon*) extract, *Eucalyptus* essential oil, *Isatis* root polysaccharides, tea seed saponins, *Tiliacora triandra* (*T. triandra*) extract, *Caesalpinia sappan* (*C. sappan*) extract, *Cryptoporus volvatus* (*C. volvatus*) extract, tea polyphenols, capsicum oleoresin, garlic botanical, and turmeric oleoresin); marine-derived natural compounds (griffithsin, iota-carrageenan, and marine sulfated polysaccharide extracts); traditional Chinese medicine formulations (modified Bazhen powder, Fuzhengjiedu San, Huashi Baidu granules, the coextract of *Curcuma phaeocaulis* (*C. phaeocaulis*) and *Astragalus membranaceus* (*A. membranaceus*)).

##### Direct inactivation

Prior to viral invasion, several agents can directly inactivate PRRSV virions. *C. dactylon* extract and *Eucalyptus* essential oil can directly inactivate virions [187]. *Isatis* root polysaccharides exhibit direct virucidal effects on viruses [188]. *Eucalyptus* essential oil improves immune recognition of the virus [189]. Essential oils possess natural volatility, a property that has potential value for reducing airborne virus transmission; however, the feasibility of their use for this purpose requires further validation in terms of virology, aerosol dynamics, and toxicology.

##### Attachment and entry

*Isatis* root polysaccharides block viral entry [188]. *T. triandra* extract also decreases viral activity by blocking viral attachment and entry [190]. Griffithsin significantly inhibited PRRSV infection *in vitro* via glycan-dependent mechanisms, targeting viral surface glycoproteins and consequently blocking viral attachment [191]. Iota-carrageenan also exerts antiviral effects by inhibiting viral attachment and entry through activation of the NF-κB pathway [192].

##### Replication

Components of *C. sappan* Linn target key viral enzymes and modulate host antioxidant pathways to interfere with PRRSV replication [190]. Its ethanolic fraction containing brazilin and brazilein is responsible for this activity [193]. *C. volvatus* extract inhibits RNA-dependent RNA polymerase (RdRp) activity, thereby blocking the synthesis of viral RNA and proteins [194,195]. Tea seed saponins downregulate host cell poly(A)-binding protein expression to inhibit viral N gene expression [196]. Tea polyphenols suppress the synthesis of viral nonstructural protein 2, regulate NF-κB activity, and downregulate the expression of TNF-α and IL-6, ultimately exerting inhibitory effects during viral infection [197]. Marine sulfated polysaccharide extracts directly inhibit early viral replication and enhance the host immune response during the later stages to achieve antiviral efficacy [198]. However, many marine bioactive compounds are present in extremely low quantities in organisms, and their content fluctuates significantly depending on species differences, collection season, and environmental factors, severely hindering their precise development. Maxing Shigan and modified Bazhen powder also restricts viral replication and consistently suppresses inflammatory responses, resulting in both antiviral and anti-inflammatory effects [199]. Fuzhengjiedu San significantly reduced the expression of the PRRSV nucleocapsid protein, inhibiting viral replication [200]. However, not all compound formulations demonstrate synergistic benefits. For instance, a compound prepared from glycyrrhizic acid and matrine was found to inhibit PRRSV replication *in vitro*, whereas it increased proinflammatory cytokine levels *in vivo* and accelerated mortality in infected pigs, indicating a lack of practical clinical value [201].

##### Release

In addition to its effects on viral attachment and entry, iota‑carrageenan inhibits viral release [192].

##### Host-directed therapy

A variety of mixtures modulate host immunity, inflammation, or cell survival. *Isatis* root polysaccharides activate the TLR4 signaling pathway by upregulating the expression of TLR4, myeloid differentiation primary response gene 88 (MYD88), and p65 to suppress PRRSV-induced inflammatory storms [188]. *C. sappan* extract promotes leukocyte proliferation, increases antibody levels, reduces lung injury, and activates the NF-κB signaling pathway to modulate immune responses [202,203]. Capsicum oleoresin, garlic botanical, and turmeric oleoresin significantly reduce the levels of virus-induced inflammatory cytokines, decrease the serum viral load, and activate immune responses [204]. The coextract of *C. phaeocaulis* and *A. membranaceus* suppresses the TLR4/ NOD-like receptor thermal protein domain-associated protein 3 (NLRP3)/IL-1β inflammatory pathway, reducing the expression of inflammatory factors and protecting target organs [205]. Fuzhengjiedu San downregulates the expression of genes such as p65, JNK, and TLR4; blocks the PI3K/Akt and tumor protein p53 (p53) pathways; and upregulates antiviral genes such as Bcl-2, thereby achieving multistage viral suppression [200,206]. Maxing Shigan indirectly induces immunomodulation through regulation of the TLR, NF-κB, and JAK-STAT pathways [207]. Huashi Baidu granules inhibited the formation of NLRP3/Caspase-1/gasderminD N-terminal fragment (GSDMD-N) inflammasome and alleviated pulmonary edema [208]. Dietary soy isoflavones increase neutrophil activity, promote memory T-cell proliferation, and accelerate anti-PRRSV antibody responses, thereby accelerating viral clearance [209]. As a dietary supplement, soy isoflavones enhance immunity and reduce infection mortality *in vivo*. However, the biological mechanisms underlying their long-term effects remain to be explored.

On the other hand, monomeric compounds have well-defined structures (see Table 5), clear antiviral mechanisms, high reproducibility, and strong translational value. Hence, these 42 monomeric compounds and their corresponding anti-PRRSV mechanisms are independently summarized in Table 5.

**Table 5.** Natural active ingredients with anti-PRRSV activity.

Name	Molecular Formula	Molecular Structure	Molecular Weight (g/mol)	Experimental Model (Cell/Animal)	Tested Range	Virus Strain	Mechanism of Action	Cite
Allicin	C_6_H_10_OS_2_	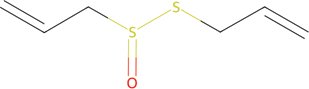	162.27	Marc-145 cells BHK-21 cells Pams	*in vitro*	PRRSV strain NL1207 (NADC30-like) PRRSV strain BLC-2 PRRSV strain TA-12	Allicin effectively blocks *in vitro* PRRSV infection by inhibiting viral entry, replication, and assembly. Concurrently, it alleviates inflammatory responses by downregulating PRRSV-induced pro-inflammatory cytokines (IFN-β, IL-6, TNF-α) and restoring the activity of the TNF and MAPK signaling pathways.	[210]
Andrographolide	C_28_h_30_knao_10_·H2O	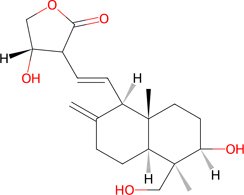	610.68	Marc-145 cells Pams	*in vitro*	PRRSV strain GD-HD PRRSV strain XH-GD PRRSV strain HNhx (NADC30-like)	Andrographolide and its derivative, potassium dehydroandrographolide succinate (PDS), significantly inhibit the replication of multiple prevalent PRRSV strains, suppress virus-induced NF-κB pathway activation and oxidative stress. PDS also directly binds to viral particles, disrupting their function.	[211]
Artesunate	C_19_H_28_O_8_	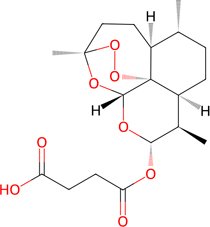	384.42	Marc-145 cells Pams	*in vitro*	PRRSV strain GD-HD PRRSV strain GD-XH PRRSV strain CH-1a PRRSV strain VR2332	Artesunate significantly inhibits PRRSV replication in cells by increasing the AMP/ADP to ATP ratio, thereby activating the AMPK-dependent Nrf2/HO-1 signaling pathway.	[212]
Astragaloside IV	C_41_H_68_O_14_	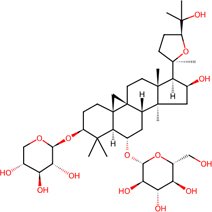	784.981	Pams	*in vitro*	PRRSV strain JL/07/SW	Astragaloside IV activates the cGAS-STING pathway-mediated type I interferon response, restores the cGAS-STING signaling activity suppressed by PRRSV infection, enhances innate immune function, promotes interferon expression, and reverses the immunosuppressive state induced by PRRSV.	[213]
Atractylodinol	C_13_H_10_O_2_	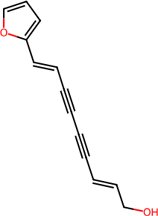	198.22	MARC-145 cells	*in vitro*	PRRSV strain YN-1	Atractylodinol interferes with viral replication and inhibits PRRSV-induced cytopathic effects by reducing the mRNA expression level of the viral ORF7 gene.	[214]
Betulonic acid	C_30_H_46_O3	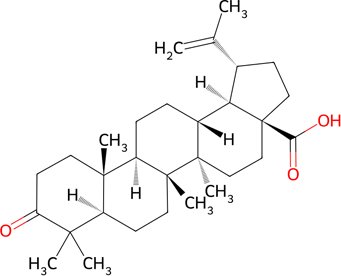	454.695	MARC-145 cells PAMs	*in vitro*	PRRSV strain GD-HD PRRSV strain CH-1a PRRSV strain NADC30-like	Betulonic acid inhibits PRRSV replication by suppressing cellular ATP production during the late stage of the viral lifecycle, specifically disrupting virion assembly/release rather than viral entry or early replication	[215]
Brazilin	C_16_H_14_O_5_	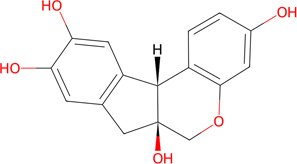	286.28	MARC-145 cells	*in vitro*	PRRSV strain VR2332	Brazilin restricts viral RNA replication by interfering with the SRCR5-CD163 interaction.	[216]
Caprylic monoglyceride	C_11_H_22_O_4_		218.29	6-week-old piglets Marc-145 cells	*in vivo* *in vitro*		Caprylic monoglyceride significantly inhibits PRRSV replication *in vitro* and *in vivo*, reduces viral load and lung lesions, modulates inflammatory cytokine balance (downregulating IL-6, IL-8, IL-1β, IFN-γ, and TNF-α; upregulating IL-10), and improves survival rates in infected hosts.	[217]
Chlorogenic acid	C_16_H_18_O_9_	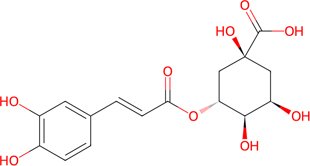	354.31	Marc-145 cells	*in vitro*	PRRSV vaccine strain JXAI-R	Chlorogenic acid exerts antiviral effects by inhibiting viral replication and directly neutralizing viral particles.	[218]
Curcumin	C_21_H_20_O_6_	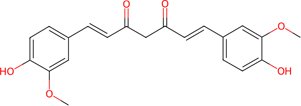	363.38	Marc-145 cells Pams	*in vitro*	PRRSV strain GD-HD PRRSV strain JXA1 PRRSV strain VR2332 PRRSV strain CH-1a	Curcumin inhibits PRRSV infection by suppressing the endocytic process of viral entry, preventing virus‒host cell membrane fusion, and maintaining cellular pH to inhibit viral internalization.	[219]
Cinnamaldehyde	C_9_H_8_O	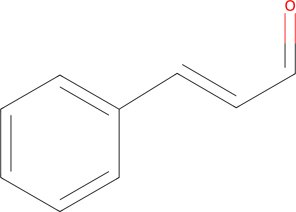	132.62	Marc-145 cells Pams	*in vitro*	PRRSV strain Li11	Cinnamaldehyde directly interacts with PRRSV particles, preventing viral attachment and entry. It inhibits PRRSV replication by disrupting the expression of dsRNA during viral replication.	[220]
Cepharanthine	C_37_H_38_N_2_O_6_	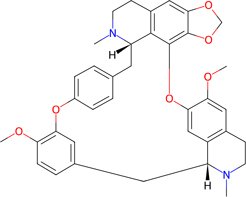	606.72	7-week-old piglets Marc-145 cells	*in vivo* *in vitro*	PRRSV strains NADC30-like	Cepharanthine blocks viral entry by directly targeting and binding to the host cell CD163 protein, a key receptor for PRRSV entry, thereby preventing interaction between the viral glycoprotein and the receptor and subsequent viral internalization.	[221]
Emodin	C_15_H_10_O_5_	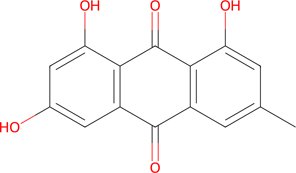	270.24	Marc-145 cells Pams	*in vitro*	PRRSV strain Li11	Emodin significantly inhibits PRRSV proliferation *in vitro* by targeting multiple stages of the PRRSV life cycle, directly inactivating viral particles, and inducing the expression of antiviral factors (IFN-α and IFN-β) via activation of TLR3.	[222]
Ethoxysanguinarine	C_22_H_19_NO_5_	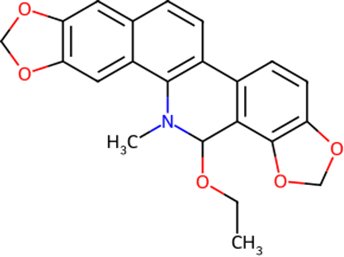	377.39	MARC-145 cells	*in vitro*	PRRSV strain YN-1	Ethoxysanguinarine interferes with the viral replication process and inhibits PRRSV-induced cytopathic effects by reducing the mRNA expression of the viral ORF7 gene.	[214]
Fangchinoline	C_37_H_40_N_2_O_6_	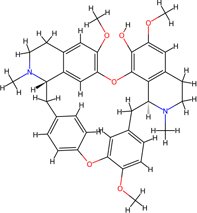	608.72	7-week-old piglets Marc-145 cells	*in vivo* *in vitro*	PRRSV strains NADC30-like	Fangchinoline blocks viral entry by directly targeting and binding to the host cell CD163 protein, a key receptor for PRRSV entry, thereby preventing interaction between the viral glycoprotein and the receptor and subsequent viral internalization.	[221]
Flavaspidic acid AB	C_12_H_26_O_8_	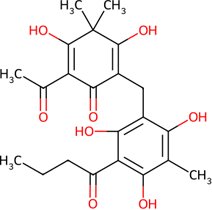	418.44	Marc-145 cells Pams	*in vitro*	PRRSV strain CH-1a HP PRRSV strain	Flavaspidic acid AB activates antiviral cytokines and inhibits viral internalization.	[223]
Formononetin	C_16_H_12_O_4_	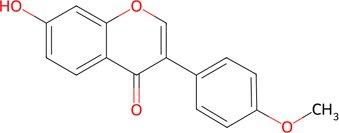	268.27	Marc-145 cells Pams	*in vitro*	PRRSV strain GSWW-15 PRRSV strain GSWW-18 PRRSV strain VR-2332	Formononetin primarily acts during the early stage of viral replication.	[224]
Germacrone	C_15_H_22_O	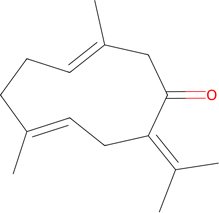	218.33	Marc145 cells Pams	*in vitro*	PRRSV strain CH-1a PRRSV strain GD-XH PRRSV strain 11FS11-GD	Germacrone significantly inhibits PRRSV proliferation by interfering with early viral replication, blocking viral RNA and protein synthesis.	[225]
Ginsenoside Rg1	C_42_H_72_O_14_	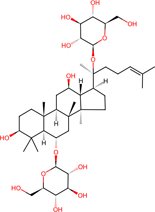	801.01	MARC-145 cells Pams 4-week-old piglets	*in vivo* *in vitro*	PRRSV strain VR2332 PRRSV strain XH-GD PRRSV strain HNLY (NADC30-like) PRRSV strain JXA1	Ginsenoside Rg1 blocks PRRSV attachment/replication/release, inhibits NF‑κB, and reduces pro‑inflammatory cytokines (IL‑1β, IL‑6, IL‑8, TNF‑α), exerting dual antiviral and immunomodulatory effects. *In vivo*, it alleviates lung injury, reduces viral load, and improves pig survival.	[226]
Glycyrrhizin	C_42_H_62_O_16_	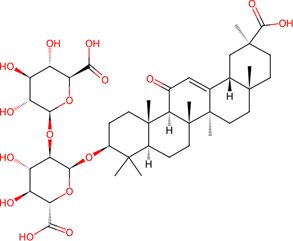	822.95	MARC-145 cells	*in vitro*	PRRSV strain WUH3	Glycyrrhizin inhibits the penetration stage of PRRSV.	[227]
Hyperoside	C_21_H_20_O_12_	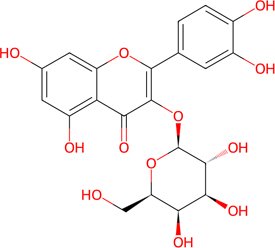	464.38	Marc-145 cells Pams 6-week-old piglets	*in vivo* *in vitro*	PRRSV-1 strains GZ11-G1 PRRSV-1 strains P073-3 PRRSV-2 strains SD16 PRRSV-2 strains CH-1a PRRSV-2 strains JXA1 PRRSV-2 strains NADC30-like	Hyperoside alleviates PRRSV-induced autophagy by inhibiting TLR4 and NF-κB to suppress pro-inflammatory cytokine upregulation and by activating the p62/Nrf2/Keap1 signaling pathway (upregulating p62, downregulating LC3-II, etc.). *In vivo*, hyperoside significantly reduces PRRSV replication in piglets.	[228]
Isobavachalcone	C_20_H_20_O_4_	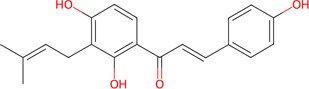	324.37	Marc-145 cells Pams	*in vitro*	PRRSV strain hun4 PRRSV strain hen-L1 (NADC30-like)	Isobavachalcone significantly inhibits *in vitro* PRRSV replication by blocking a postentry stage of PRRSV infection.	[229]
Luteolin	C_15_H_10_O_6_	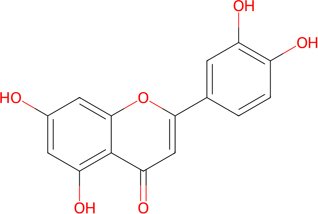	286.24	Marc-145 cells	*in vitro*	PRRSV-2 strain AHNO	Luteolin inhibits PRRSV proliferation by inducing an innate antiviral response via upregulation of type I and type II IFNs (increasing IFN-α, IFN-β, IFN-γ, and IL-10 expression and downregulating TNF-α expression).	[230]
Lycopene	C_40_H_56_	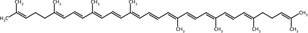	536.88	Marc145 cells HEK293T cells	*in vitro*	PRRSV-GFP strain PRRSV strain HN07-1	Lycopene inhibits PRRSV replication by modulating ROS levels and alleviating inflammatory responses.	[231]
Lycorine hydrochloride	C_16_h_18_clno_4_	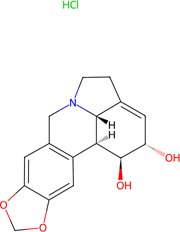	323.77	Marc145 cells Pams	*in vitro*	PRRSV-2 strains GD-HD PRRSV-2 strains CH-1a PRRSV-2 strains NADC30-like hnhx	Lycorine hydrochloride reduces PRRSV replication by rapidly upregulating the p38 MAPK signaling pathway, increasing the expression of NLRP3 and Caspase-1 proteins and thereby promoting the conversion of pro-IL-1β to mature IL-1β.	[232]
Magnolol	C_18_H_18_O_2_	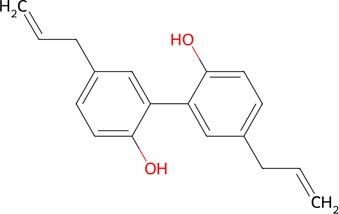	266.34	Marc145 cells	*in vitro*	PRRSV strain CHR6	Magnolol inhibits PRRSV replication by upregulating the expression of A Disintegrin And Metalloprotease 17 (ADAM17). It also increases the expression of key cytokines, including IL-6, IL-8, and TNF-α, thereby enhancing the host cell’s effective antiviral capacity.	[233]
Mannan oligosaccharide	C_34_H_58_N_2_O_26_		456.68	3-week-old pig	*in vivo*	PRRSV strain P-129	Mannan oligosaccharides (MOS) enhance the host’s resistance to PRRSV and mitigate the impact of the virus on reproductive performance by increasing white blood cell and lymphocyte counts and inhibiting the expression of inflammatory factors such as TNF-α.	[234]
Matrine	C_15_H_24_N_2_O	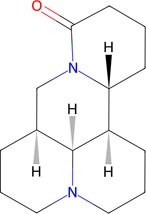	248.36	Marc-145 cells Pams 6-week-old KM mice,	*in vitro*	PRRSV vaccine strain JXAI-R PRRSV strain JS-1	Matrine directly inhibits viral N protein synthesis and blocks caspase‑3‑mediated apoptosis, providing dual regulation. Against PRRSV/PCV2 coinfection, it targets HSPA8/HSP90AB1 to suppress replication, modulates TLR3/4‑NF‑κB to reduce cytokines (TNF‑α), and enhances macrophage phagocytosis and lymphocyte proliferation.	[176,235–237]
Mogroside V	C_60_H_102_O_29_	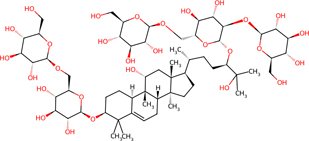	1287.45	Marc-145 cells Pams	*in vitro*	PRRSV strain NADC30 HP PRRSV strain	Mogroside V exerts dual antiviral effects by directly inhibiting PRRSV replication and upregulating the gene expression of immunomodulatory cytokines (IL-1, IL-2, IL-8, and IL-18).	[238]
Naringenin	C_15_H_12_O_5_	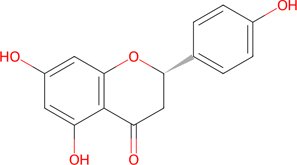	272.25	Marc-145 cells	*in vitro*	PRRSV strain JL/07/SW	Naringenin ameliorates PRRSV-induced innate immunosuppression by modulating the RIG-I-MAVS signaling pathway, significantly upregulating the expression of antiviral cytokines (IFN-I, IL-10, and ISGs) that are suppressed during the late stage of PRRSV infection.	[239]
Neferine	C_38_H_44_N_2_O_6_	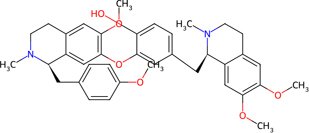	624.78	Marc-145 cells	*in vitro*	PRRSV strain CHR6	Neferine inhibits PRRSV infection and replication by inhibiting p65 nuclear translocation.	[240]
Platycodin D	C_57_H_92_O_28_	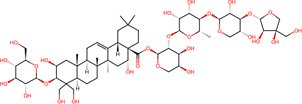	1225.33	Marc145 cells, Pams	*in vitro*	PRRSV strain CH-1a PRRSV strain VR2332 PRRSV strain GD-HD PRRSV strain GD-XH	Platycodin D inhibits PRRSV invasion and release by acting directly on viral particles and reduces the production of PRRSV-induced inflammatory cytokines (IFN-β, IL-1α, IL-6, IL-8, and TNF-α).	[241]
Proanthocyanidin A2	C_30_H_24_O_12_	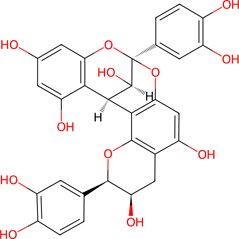	576.51	Marc145 cells Pams	*in vitro*	PRRSV strain CH-1a PRRSV strain GD-HD PRRSV strain GD-XH	Proanthocyanidin A2 blocks viral entry and release and significantly inhibits the expression of PRRSV-induced cytokines (e.g. TNF-α, IFN-α, IL-1β, and IL-6).	[242]
Rottlerin	C_30_H_28_O_8_	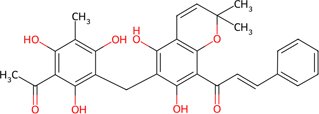	516.55	10-week-old piglets Marc-145 cells	*in vivo* *in vitro*	PRRSV strain LMY	Rottlerin inhibits PRRSV replication by suppressing the PKCδ-Cofilin signaling pathway to reduce actin polymerization, thereby blocking the macropinocytosis and endocytosis entry pathways of PRRSV. Ultimately, it reduces blood viral load and alleviates interstitial pneumonia symptoms in infected pigs.	[243,244]
Sanggenon C	C_40_H_36_O_12_	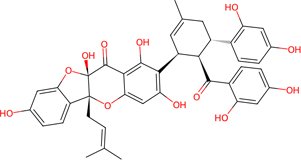	708.71	35-day-old pig Marc-145, CRL-2843, Vero PK-15 HEK-293T cellspams	*in vivo* *in vitro*	PRRSV strain SD-YL1712	Sanggenon C significantly alleviates clinical symptoms in PRRSV-infected piglets, reduces viral load in lungs and blood, mitigates lung injury, inhibits inflammatory cytokine secretion, and shortens the virus shedding period. SC inhibits PRRSV replication by promoting TRAF2 expression to inhibit the activation of the NF-κB signaling pathway.	[245,246]
Sanguinarine	C_20_H_14_NO_4_	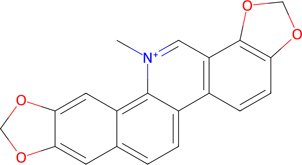	332.35	Marc-145 cells	*in vitro*	PRRSV strain WUH3	Sanguinarine inhibits viral proliferation by targeting the internalization, replication, and release stages of PRRSV, with key targets including ALB, AR, MAPK8, MAPK14, IGF1, GSK3B, PTGS2, and NOS2. Its combination with chelerythrine enhances the antiviral effect.	[247]
Scutellarin	C_21_H_18_O_12_	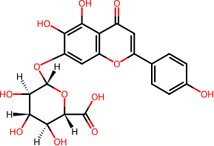	446.36	Marc-145 cells Pams	*in vitro*	PRRSV vaccine strain JXAI-R PRRSV strain GD-HD Highly pathogenic NADC30-like- PRRSV strain VR2332 PRRSV strain SD16	Scutellarin inhibits *in vitro* PRRSV infection by directly binding viral particles to interfere with the replication cycle, inhibiting host receptor (CD151 and CD163) expression to block viral entry, downregulating PRRSV-induced pro-inflammatory cytokines (IFN-α, IL-6, IL-8, and TNF-α), and modulating anti-inflammatory immune responses.	[218,248]
Sodium tanshinone IIA	C_19_H_17_O_6_S	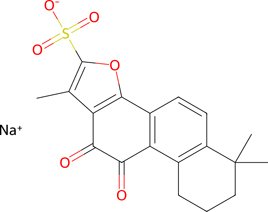	396.39	Marc145 cells	*in vitro*	PRRSV vaccine strain JXAI-R	Sodium tanshinone IIA dose-dependently inhibits PRRSV replication by directly inactivating viral particles and inhibiting the replication stage (including blocking N protein expression and virus-associated autophagy genes Beclin1, ATG5, and ATG7), while also alleviating virus-induced apoptosis.	[249–251]
Toosendanin	C_30_H_38_O_11_	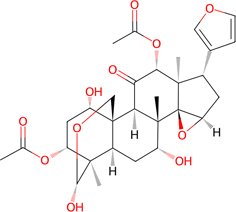	574.62	Marc145 cells Pams	*in vitro*	PRRSV strain CH-1a PRRSV strain VR2332 PRRSV strain hnhx(NADC30-like)	Toosendanin significantly inhibits PRRSV replication by upregulating IFI16 expression, activating caspase-1, and inducing IL-1β maturation.	[252]
Ursonic acid	C_30_H_46_O_3_	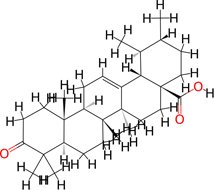	454.69	Vero cells Marc-145 cells ST cells HEK-293 T cells Pams	*in vitro*	PRRSV strain S1 PRRSV strain FJ1402 (NADC30-like) PRRSV strain CH-1a PRRSV strain VR2332 PRRSV strain GD-XH	Ursonic acid inhibits PRRSV replication and reduces IFN-β production by targeting PTPN1 and inhibiting its phosphatase activity.	[253,254]
Xanthohumol	C_21_H_22_O_5_	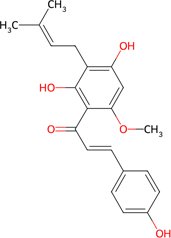	354.40	Marc-145 cells Pams 5-week-old piglets	*in vivo* *in vitro*	PRRSV strain BB0907 PRRSV strain S1 PRRSV strain FJ1402 (NADC30-like)	Xanthohumol synergistically inhibits viral proliferation by blocking PRRSV attachment and invasion and activating the Nrf2-HMOX1 antioxidant pathway while downregulating pro-inflammatory cytokine expression (IL-1β, IL-6, IL-8, and TNF-α). It alleviates clinical symptoms, lung pathological damage, and inflammatory responses in PRRSV-infected pigs.	[255,256]
(-)-Epigallocatechin-3-gallate	C_22_H_18_O_11_	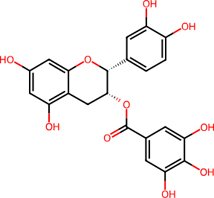	458.37	Marc-145 cells Pams	*in vitro*	PRRSV strain HN07-1 PRRSV strain BJ-4 PRRSV strain rhp-PRRSV/2006	(-)-Epigallocatechin-3-gallate binds viral particles to block attachment; downregulates host receptors (CD163, MYH9, HS); blocks replication/assembly; inhibits lipid droplet formation, metabolic reprogramming, and autophagy‑dependent proliferation; and reduces pro‑inflammatory cytokines (TNF‑α, IL‑6, IL‑8).	[257,258]

Note: The chemical structures mentioned in the table are from https://www.chemspider.com/.

#### Effects of natural compounds against PRV

The natural compounds with demonstrated anti-PRV activity primarily include crude plant extracts and microbial metabolites. Plant extracts (fermented *Erigeron breviscapus* (*E. breviscapus*) flavonoids, *Houttuynia cordata* (*H. cordata*) extract, *Morus alba* L. extract, *Glycyrrhiza* polysaccharide, *Cistanche deserticola* (*C. deserticola*) polysaccharides, *Hippophae rhamnoides* (*H. rhamnoides*) polysaccharides, *Lysimachia christinae* (*L. christinae*) polysaccharides, *Isatis indigotica* (*I. indigotica*) extract, *Rehmannia glutinosa* (*R. glutinosa*) polysaccharide, *Taraxacum mongolicum* (*T. mongolicum*) (dandelion) extract, *Sasa veitchii* (*S. veitchii*) extract, *Panax notoginseng* (*P. notoginseng*) polysaccharides, *P. grandiflorus* polysaccharides, *Kaempferia galanga* L. rhizome and *P. hydropiper* L. extract, and the compound carvacrol); microbial metabolites include napyradiomycin A4 and metabolites of *Bacillus subtilis* L2.

##### Direct inactivation

Before PRV comes into contact with cells, the injection of fermented *E. breviscapus* flavonoids and *H. cordata* polysaccharide directly inactivates free virions [259,260]. *Radix isatidis* polysaccharide has both direct virucidal and prophylactic effects [261].

##### Attachment and entry

*Morus alba* L. extract and *Glycyrrhiza* polysaccharide shield cellular receptors to block viral attachment and internalization [262,263]. *C. deserticola* polysaccharides inhibit membrane fusion or endocytic pathways to reduce viral entry. *H. rhamnoides* polysaccharides and *L. christinae* polysaccharides suppress virus–receptor binding and attenuate viral invasion [264–266]. *I. indigotica* extracts and *R. glutinosa* polysaccharide both interfere with gB glycosylation and gB/gD-mediated attachment [267,268]. Secondary metabolites of *Bacillus subtilis* L2 also prevent viral entry but also affect attachment [269].

##### Replication

Following viral entry into cells, dandelion aqueous extract and *S. veitchii* extract rapidly downregulate the expression of key viral genes such as *IE180*, *EP0*, *UL29*, *UL44*, and *UL52*, thereby inhibiting both viral attachment and viral replication [270]. Polysaccharides from *P. notoginseng* concurrently activate immune and antioxidant pathways to interfere with viral replication [271]. *P. grandiflorus* polysaccharides activate the Akt/mechanistic target of rapamycin (mTOR) pathway to inhibit virus-induced autophagy, thereby restricting intracellular viral amplification [272]. Napyradiomycin A4 derived from *Streptomyces kebangsaanensis* WS-68302 inhibits viral protein synthesis [273]. *Bacillus subtilis* L2 metabolites continue to suppress viral replication and alleviate encephalitis and pneumonia symptoms [269].

##### Host-directed therapy

These polysaccharides also alleviate PRV-induced mitochondrial damage by maintaining mitochondrial membrane potential, improving mitochondrial morphology and suppressing mitochondrial apoptosis by downregulating BCL2-associated X (Bax) expression and upregulating B-cell lymphoma-2 (Bcl-2) expression, which protects host cells from PRV-induced apoptosis [274]. Fermented *E. breviscapus* flavonoids modulate the TLR4/MyD88/NF-κB pathway, promote microglial M2 polarization, and balance inflammatory responses, which collectively contribute to the anti-PRV effect [260]. Dandelion aqueous extract and *Kaempferia galanga* L. rhizome reduce the tissue viral load, mitigate pathological injury, and restore the Th1/Th2 balance by modulating IFN-α/γ and IL-4 levels [275,276]. Oregano essential oil (containing carvacrol) increases the expression of IFN-β/γ, TNF-α, and IL-12 while inhibiting apoptosis [277]. The ethyl acetate fraction of flavonoids from *P. hydropiper* L. blocks IκBα phosphorylation, p65 nuclear translocation, and p38/ extracellular signal‑regulated kinase (ERK) phosphorylation, thereby inhibiting both the NF-κB and MAPK pathways and comprehensively alleviating PRV-associated inflammation [278].

Unlike crude plant extracts or polysaccharide mixtures, antimicrobial peptides are a class of natural monomeric compounds composed of specific amino acid sequences. They possess defined primary structures and exhibit antiviral activity as individual molecular entities. The antimicrobial peptides piscidin, caerin, and maculatin and the chicken-derived peptide Cathelicidin-B1(CATH-B1) disrupt the ability of PRV capsids to block viral entry [279,280]. CATH-B1 also inhibits viral absorption and activates the TLR4/JNK/IRF3 axis to enhance host immunity [279], whereas piscidin, caerin, and maculatin suppress PRV-induced apoptosis, thereby reducing tissue damage and mortality in mice [280]. Collectively, these AMPs exert multilayered protection against PRV without relying on a single small-chemical scaffold.

In addition to these peptide-based monomers, a series of well-defined small-molecule monomeric compounds, exemplified by the 20 entities cataloged in Table 6, feature unambiguous chemical structures, straightforward mechanistic interpretation, improved experimental reproducibility, and superior translational potential. Accordingly, these compounds and their respective anti-PRV mechanisms are compiled separately in Table 6.

**Table 6.** Natural active ingredients with anti-PRV activity.

Name	Molecular Formula	Molecular Structure	Molecular Weight (g/mol)	Experimental Model (Cell/Animal)	Tested Range	Virus Strain	Mechanism of Action	Cite
Berbamine	C_37_H_40_N_2_O_6_	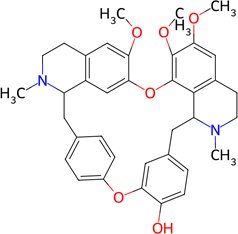	608.735	Vero cells BALB/c mice	*in vivo* *in vitro*	PRV strain JSY13	Berbamine inhibits PRV proliferation by targeting the replication and release stages of the viral life cycle. In experiments with artificially PRV-infected mice, berbamine significantly alleviated clinical symptoms and brain histopathological changes induced by PRV infection.	[281]
Curcumin	C_21_H_20_O_6_	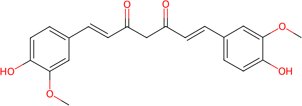	363.38	PK-15 cells PC-12 cells Sprague Dawley rats (<24 h)	*in vivo* *in vitro*	PRV strain Rong A	Curcumin exerts anti‑PRV neuroprotection via BDNF/TrkB (reducing oxidative stress, mitochondrial dysfunction, apoptosis). It also upregulates miR‑155‑5p to inhibit AAK1, leading to Numb upregulation and Notch2 suppression, thereby blocking the PRV lytic cycle and reactivation.	[282,283]
Dihydromyricetin	C_15_H_12_O_8_	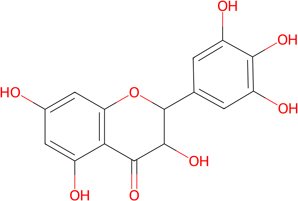	320.25	PK-15 cells	*in vitro*	PRV strain HLJ	Dihydromyricetin interferes with PRV entry into host cells and inhibits pyroptosis by downregulating the expression of pyroptosis-related genes (Caspase-1, GSDMD, IL-1β, and IL-18).	[284]
Isobavachalcone	C_20_H_20_O_4_	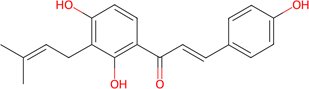	324.37	PK-15 cells RK13 cells HEK293 cells	*in vitro*	/	Isobavachalcone blocks the virus-mediated cell‒cell fusion process.	[285]
Emodin	C_15_H_10_O_5_	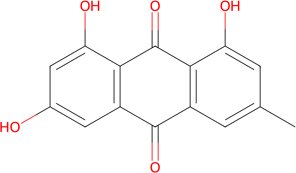	270.24	PK-15 cells KM mice	*in vivo* *in vitro*	PRV strain TJ	Emodin exerts direct antiviral effects by inhibiting PRV gene transcription, interfering with gB/gD protein function, and blocking the viral replication phase. It also achieves dual anti-inflammatory and immunomodulatory effects by modulating the TNF-α/IFN-γ/IL-4 balance to regulate the Th1/Th2 immune response and intervening in host apoptotic pathways.	[286]
Gallocatechin gallate	C_22_H_18_O_11_	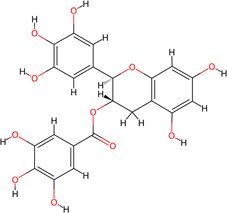	458.37	PK-15 cells	*in vitro*	PRV strain JSY13	Gallocatechin gallate blocks the PRV replication cycle by inhibiting the viral entry and release stages, with its inhibitory effect being dose dependent.	[287]
Ginkgolic acid	C_22_H_34_O_3_	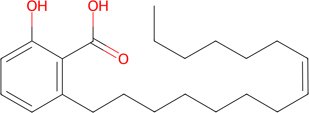	346.51	PK15 cells Vero cells	*in vitro*	PRV strain JSY13	Ginkgolic acid specifically targets the late gene transcription stage of the PRV replication cycle, blocking the synthesis of viral structural proteins.	[288]
Luteolin	C_15_H_10_O_6_	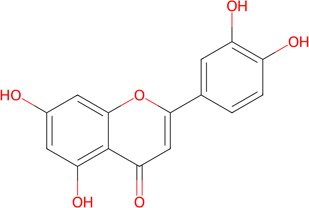	286.24	PK15 cells BALB/c mice	*in vivo* *in vitro*	PRV strain ZJ01	Luteolin blocks viral replication by directly inhibiting PRV mRNA and gB protein expression, while upregulating SOD/GSH antioxidant capacity and modulating immune balance (increasing IFN-γ, decreasing TNF-α/IL-4). In mouse models, it significantly delayed disease onset, reduced organ viral load, and improved survival rates.	[289]
Myricetin	C_15_H_10_O_8_	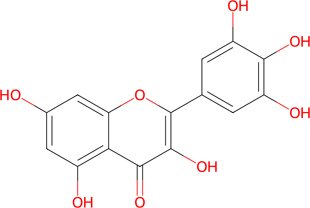	318.24	PK15 cells KM mice	*in vivo* *in vitro*	PRV strain Ra	Myricetin inhibits PRV replication by activating the cGAS/STING-IRF3 and JAK/STAT pathways to enhance the type I interferon response (upregulating IRF3, enhancing STAT1 phosphorylation).	[290]
Naringenin	C_15_H_12_O_5_	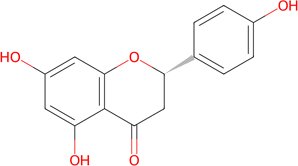	272.25	PK15 cells KM mice	*in vivo* *in vitro*	PRV strain TJ	Naringenin significantly inhibits PRV replication in the brain, lungs, and kidneys of mice *in vivo* and alleviates PRV-induced pathological changes by inhibiting the TLR4/NF-κB signaling pathway (downregulating TLR4 and P65 protein expression, reducing inflammatory cytokine release) and directly targeting the PRV gB protein (blocking the viral replication stage).	[291]
Quercetin	C_15_H_10_O_7_	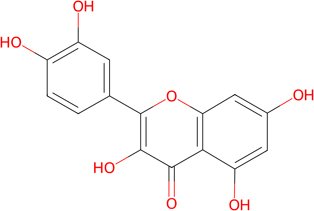	302.24	Vero cells PK-15 cells 3D4/2 cells BALB/c mice	*in vivo* *in vitro*	PRV strain HNX PRV strain Bartha PRV strain Ea, PRV strain Fa	Quercetin inhibits viral attachment and invasion by directly blocking the binding of the PRV gD protein to the host Nectin-1 receptor. It also reduces ROS generation by modulating the TXNIP/NOS2-related lncRNA/miRNA network, effectively alleviating oxidative stress damage while significantly reducing tissue viral load.	[292,293]
Quercitrin	C_21_H_20_O_11_	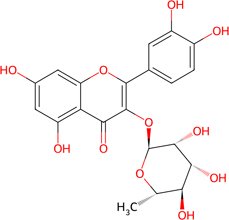	448.38	3D4/2 cells	*in vitro*	/	Quercitrin alleviates oxidative stress and modulates epigenetic modifications by inhibiting PI3K/AKT pathway activation, activating the AMPK/Nrf2 and PPAR-γ/Nrf2 signaling axes, and upregulating antioxidant enzymes (HO1, NQO1) and histone acetylation (AcH3, AcH4), while simultaneously reducing virus-induced ROS production.	[294]
Resveratrol	C_14_H_12_O_3_	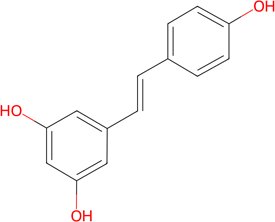	228.25	PK-15 cells 28-day-old healthy pigs KM mice	*in vivo* *in vitro*	PRV strain Rong A PRV strain Ra PRV strain Bartha-K61	Resveratrol reduces viral load, pathology, and mortality via: reversing PRV immunosuppression (OPN–ERK/JNK–IL‑1β), blocking NF‑κB (inhibiting IκBα degradation and RelA nuclear translocation), targeting IE180 to suppress replication, and elevating immune factors (IFN‑γ, IL‑12, TNF‑α, IFN‑α). It alleviates encephalitis by modulating microglial polarization and mitigates reproductive disorders by maintaining progesterone levels.	[291,295–300]
Rosmarinic acid	C_18_H_16_O_8_	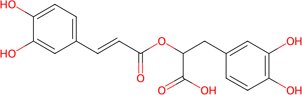	360.31	PK-15 cells ICR mice	*in vivo* *in vitro*	PRV strain Ra	Rosmarinic acid inhibits PRV *in vitro* and *in vivo*, alleviates tissue damage, and improves survival by activating the cGAS‑STING pathway to upregulate IFN‑β, thereby blocking PRV attachment, penetration, and intracellular replication. It also reduces inflammatory cytokines (TNF‑α, IL‑1β, IL‑6) and pro‑apoptotic factors (Caspase‑3, Bax) while increasing anti‑apoptotic Bcl‑2.	[301]
(S)-10-Hydroxycamptothecin	C_20_H_16_N_2_O_5_	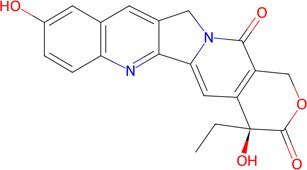	364.35	BHK-21 cells N2a cells HEK-293 T cells	*in vitro*	PRV strain ZJ01 PRV strain LA PRV strain Bartha-K61	(S)-10-Hydroxycamptothecin blocks PRV genome replication by targeting DNA topoisomerase I (TOP1).	[302]
Salvianolic Acid A	C_26_H_22_O_10_	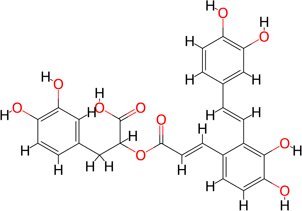	494.45	Vero cells LLC-PK1 cells HT22 cells KM mice	*in vivo* *in vitro*	PRV strain QXY PRV strain HN	Salvianolic acid A inactivates PRV by directly disrupting the viral envelope structure and inhibits viral attachment and internalization.	[303]
Scutellarin	C_21_H_18_O_12_	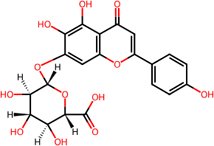	446.36	PK15 cells	*in vitro*	PRV strain henlh/2017 PRV-GFP strain	Scutellarin downregulates 14 PRV proteins involved in replication, release, and immune evasion. It reduces ROS and mitochondrial abnormalities (via NFU1 inhibition), activates complement/coagulation cascades (F3 upregulation), and enhances nucleocytoplasmic function (SLC3A2/CEBPB upregulation), thereby restoring host antiviral defense.	[304]
Piceatannol	C_14_H_12_O_4_	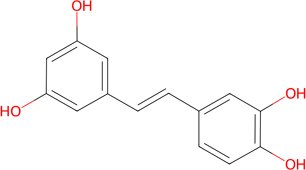	244.24	PK-15 cells KM mice	*in vivo* *in vitro*	PRV strain TJ	Piceatannol exerts antiviral effects *in vitro* by inhibiting viral attachment and replication and reducing apoptosis. *In vivo*, it reduces tissue viral load and modulates the immune response, thereby delaying disease onset and improving survival rates.	[305]
Kaempferol	C_15_H_10_O_6_	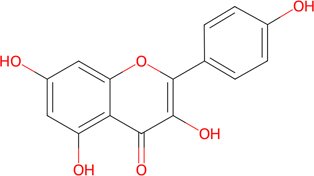	286.24	PK-15 cells KM mice	*in vivo* *in vitro*	PRV strain Rong A	Kaempferol directly inhibits viral replication, blocks the IE180-mediated transcriptional cascade, and modulates host immunity (IL-1β, IL-6, IFN-γ). It enhances the early inflammatory response to reduce tissue viral load and avoids immunopathological damage in later stages.	[306]
(−)-Epigallocatechin-3-gallate	C_22_H_18_O_11_	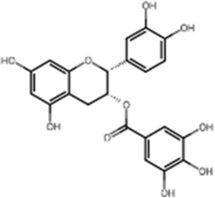	458.37	PK15 B6 Vero cells BALB/c mice	*in vivo* *in vitro*	PRV strain Ra PRV strain XJ5	(-)-Epigallocatechin-3-gallate exhibits significant antiviral activity by reducing the expression of viral proteins (such as gE and UL42), inhibiting intracellular PRV replication as well as viral attachment and entry.	[307]

Note: The chemical structures mentioned in the table are from https://www.chemspider.com/.

### Effects on bovine viral reproductive disorders

#### Effects of natural compounds against BoHV-1

This section summarizes natural compounds with anti-BoHV-1 activity; these compounds can be divided into mixtures (extracts from *Trichilia catigua* (*T. catigua*) bark, *Phragmites australis* (*P. australis*), *Desmodium canadense* (*D. canadense*), *Gigartina skottsbergii* (*G. skottsbergii*), and *Lentinula edodes* (*L. edodes*); polysaccharides from *Azadirachta indica* (*A. indica*) leaves; and monomeric compounds (kievitone, banana lectin, Meth O, curcumin, kaempferol, and 3-O-methylfunicone).

##### Direct inactivation

Prior to BoHV-1 invasion, the extract of *Thymbra spicata* L. directly inactivates virions [308]. Extracts from *T. catigua* bark, *P. australis*, and *D. canadense* directly disrupt viral integrity [309–311]. *D. canadense* was used as animal feed by early American settlers and is also commonly consumed by wild herbivores and livestock, indirectly indicating its relatively high degree of safety.

##### Attachment and entry

*L. edodes* extract, polysaccharides from *A. indica* leaves, and λ-carrageenan extracted from *G. skottsbergii* disrupt the viral envelope or physically block attachment sites, providing a first line of defense [312–314]. Native banana lectin blocks membrane contact by masking glycosylation sites via its mannose-binding domain [315]. The active component Meth O from *Schisandra chinensis* inhibits viral endocytosis by blocking the PI3K/Akt pathway and restricts viral entry and progeny dissemination by increasing the methylation (m6A) of gD [316]. Curcumin inhibits BoHV-1 entry by modulating lipid raft formation [317]. *In vitro* experiments have confirmed that *Lablab purpureus* exhibits inhibitory activity against BoHV-1. Through computational simulation, kievitone was identified as a key active component that may inhibit viral attachment and membrane fusion by interfering with the binding of gD to host receptors [318]. Notably, this mechanism is currently based solely on in silico predictions and has not yet been validated by other experimental approaches.

##### Replication

The extract of *Thymbra spicata* L. inhibits BVDV replication [308], and kaempferol targets and inhibits the Akt signaling pathway, thereby blocking late-stage viral replication [319]. Additionally, the metabolic products of *Mannheimia haemolytica* inhibit BoHV-1 replication by acidifying the microenvironment, while the fungal metabolite 3-O-methylfunicone suppresses BoHV-1 replication and protects host cells via the aryl hydrocarbon receptor pathway [320,321]. However, all the currently mentioned natural compounds with anti-BoHV-1 activity have been investigated only *in vitro*, and *in vivo* data are lacking. Consequently, their actual efficacy, safety, and dosage regimens in animals remain to be validated, and there is still a considerable gap before they can be applied in veterinary clinical practice.

#### Effects of natural compounds against BVDV

This section summarizes several natural compounds with demonstrated antiviral activity against BVDV, including *Thymbra spicata* L. extract, *Codonopsis pilosula* (C. *pilosula*) polysaccharides, metabolites of *Bacillus* sp., banana lectin, *Petiveria alliacea* (*P. alliacea*), *Salvia officinalis* (*S. officinalis*), various plant essential oils, *Glycyrrhiza* polysaccharide, leaf saponins of *Quillaja brasiliensis* (*Q. brasiliensis*), and monolaurin. Notably, most of these compounds lack well‑defined monomeric components, and their actual active ingredients remain unknown, which severely limits their pharmacological reproducibility and clinical translation.

##### Direct inactivation

Prior to viral entry, extracts of *Thymbra spicata* L. and polysaccharides from *C. pilosula* directly inactivate viral particles [308,322].

##### Attachment and entry

Metabolites from marine *Bacillus* sp. and polysaccharides from *C. pilosula* can block viral absorption [323]. Banana lectin, by binding to envelope glycoproteins and interfering with host membrane complexes, disrupts viral entry [315]. *In vitro* experiments fail to reflect the impact of the actual formulation, whereas the direct injection of banana lectin induces marked inflammatory and immune responses, leading to significant adverse effects [324].

##### Replication

Following cellular entry, extracts of *Thymbra spicata* L. and the terpenoid-polyphenol fraction of *P. alliacea* inhibit viral replication [308,325]. *S. officinalis* interferes with the entire viral replication cycle [326]. Essential oils from various plants – including basil, lemon, and citronella – as well as their active components can directly disrupt the viral lipid envelope or interfere with viral replication [327–329].

##### Host-directed therapy

*Glycyrrhiza* polysaccharide, via its unique uranyl sulfate groups, activates the IRF-1/IRF-3 signaling pathway and enhances innate immune responses [330]. Leaf saponins from *Q. brasiliensis* promote BVDV-specific antibody and Th1/CD8^+^ T-cell responses and, when used as an adjuvant, can induce a mixed Th1/Th2 immune response [331,332]. Monolaurin increases the efficacy of BVDV vaccines by suppressing the production of proinflammatory cytokines and modulating T-cell proliferation [333]. The above substances act only as vaccine adjuvants to combat viruses by enhancing immune responses; whether they possess direct antiviral activity remains unclear.

Unlike the abovementioned mixtures with complex and variable compositions, well‑defined monomeric compounds offer unambiguous structural identification, reliable quality control, and straightforward safety evaluation. Hence, they are summarized independently in Table 7, which details 15 natural anti‑BVDV agents with confirmed monomeric structures and their mechanisms of action.

**Table 7.** Natural active ingredients with anti-BVDV activity.

Name	Molecular Formula	Molecular Structure	Molecular Weight (g/mol)	Experimental Model (Cell/Animal)	tested Range	Virus Strain	Mechanism of Action	Cite
Artemisinin	C_15_H_22_O_5_	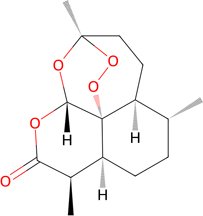	282.34	Model bovine epithelial cells from embryonic trachea	*in vitro*	BVDV strain C24V	Artemisinin targets the BVDV replication stage via the iron-dependent free radical release mechanism of its endoperoxide bridge, inhibiting virion production. It acts synergistically with interferon-α and ribavirin to alleviate immunosuppression and organ damage.	[334,335]
Berbamine hydrochloride	C_37_H_41_ClN_2_O_6_	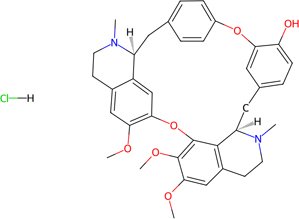	645.19	MDBK cell	*in vitro*	NCP BVDV strain BJ175170	Berbamine hydrochloride inhibits BVDV replication, particularly during viral attachment and release stages, by blocking autophagosome-lysosome fusion through the inhibition of LC3-LAMP1 binding, thereby interfering with the late stage of autophagy.	[336]
Bergamottin	C_21_H_22_O_4_	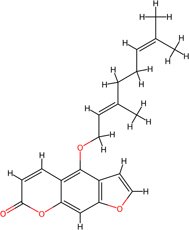	338.4	MDBK cell 6–8 weeks female BALB/c mice	*in vivo* *in vitro*	BVDV strain NADL	Bergamottin exerts anti-BVDV effects by inhibiting viral replication and release, and suppressing the ROS-ER stress-apoptosis axis (downregulating GRP78, PERK, eIF2α, ATF4, and CHOP expression; modulating Bcl-2/Bax/caspase-3 expression). It effectively reduces viral load and tissue damage.	[337]
Curcumin	C_21_H_20_O_6_	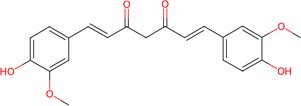	368.38	MDBK cell BALB/c mice	*in vivo* *in vitro*	BVDV strain NADL	Daidzein blocks viral attachment, while curcumin inhibits the virus across multiple stages and modulates lipid metabolism; both compounds alleviate BVDV-induced immunosuppression and organ damage.	[334]
Daidzein	C_15_H_10_O_4_	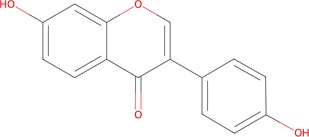	254.23					
Ethyl gallate	C_9_H_10_O_5_	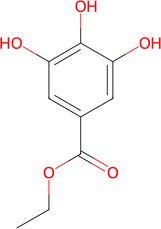	198.17	MDBK cell	*in vitro*	BVDV strain BJ175170	Ethyl gallate blocks the early stages of BVDV infection (postattachment entry and replication) by promoting IFITM3 expression (inhibiting viral membrane fusion and endosomal escape), enhancing lysosomal acidification, and protease activity (increasing cathepsin B levels). It also inhibits BVDV‑induced autophagic flux by upregulating LC3‑II and p62.	[338]
Forsythiaside A	C_29_H_36_O_15_	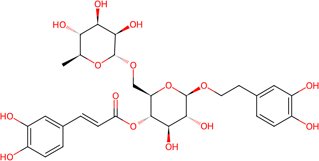	624.59	MDBK cell 6–8 weeks female BALB/c mice Bovine pbmcs	*in vivo* *in vitro*	BVDV strain BJ175170 BVDV strain C24V	Forsythiaside A directly inhibits viral replication and employs a dual immunomodulatory mechanism: it enhances T-cell activation via the TRAF2-CD28/4-1BB pathway (upregulating CD28, 4-1BB, Bcl-xL; downregulating Bim) and promotes PBMC proliferation, while balancing cytokines. Concurrently, it regulates innate immunity by inhibiting the NLRP3 inflammasome and activating type I interferons. As a vaccine adjuvant, it further reduces splenic viral load and alleviates histopathological damage by promoting CD4+/CD8+ T-cell responses and neutralizing antibody production.	[339,340]
Gypenoside	C_13_H_16_O_5_	/	268.26	MDBK cell	*in vivo* *in vitro*	BVDV strain BJ175170	Gypenoside inhibits BVDV replication by interfering with viral attachment and internalization and by inducing apoptosis in infected cells.	[341]
Icariin	C_33_H_40_O_15_	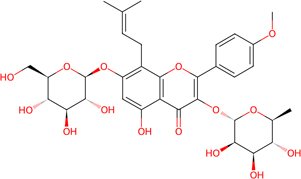	676.66	MDBK cell	*in vitro*	/	Matrine and icariin inhibit BVDV through multiple pathways: exerting direct virucidal effects, blocking viral adsorption, and activating the IFN-I pathway via the RIG-I/TLR3/IRF3 axis.	[342]
Matrine	C_15_H_24_N_2_O	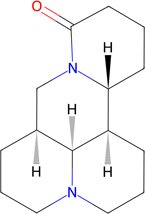	248.36					
Melatonin	C_13_H_16_N_2_O_2_	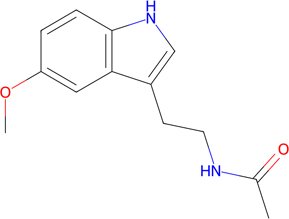	232.27	MDBK cell BALB/c mice	*in vivo* *in vitro*	BVDV strain NADL BVDV 1 strain NADL	Melatonin indirectly regulates autophagy initiation via the ER stress/PERK/eIF2α/ATF4 signaling axis and inhibits the NF-κB pathway (reducing the p-IκBα/IκBα and p-p65/p65 phosphorylation ratios). It also blocks autophagic degradation by upregulating LC3-II and p62 protein levels, thereby reversing BVDV-induced autophagic flux. It coordinately inhibits BVDV replication, alleviates inflammation, and blocks viral invasion by upregulating antiviral factors and downregulating tight junction proteins.	[343,344]
Oleandrin	C_32_H_48_O_9_	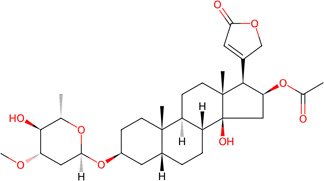	576.72	MDBK cell	*in vitro*	BVDV vaccine strain Singer (Lot# 040917)	Oleandrin acts against BVDV by inhibiting Na^+^/K^+^-ATPase, blocking endoplasmic reticulum stress, and inhibiting viral protein synthesis and progeny virion envelope formation; it is effective when administered preinfection or early within 12 hours post-infection.	[345]
Phlorizin	C_21_H_24_O_10_	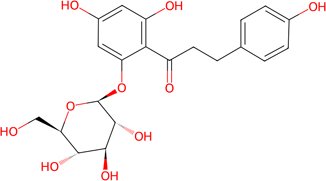	436.41	BALB/c mice MDBK cell	*in vivo* *in vitro*	Cp BVDV strain NADL CP BVDV-1a strain NADL NCP BVDV-1b strain NY-1 NCP BVDV-2 isolate strain YNJG2020	Phlorizin exerts anti‑BVDV effects by targeting autophagy (downregulating Beclin‑1 and LC3B‑II), inhibiting viral replication, modulating IFN‑I signaling, and balancing immunity (upregulating RIG‑I; downregulating TLR3 and NLRP3). In vivo, it reduces viral load and pathology. It also suppresses BVDV replication by restoring T‑lymphocyte ratios and remodeling gut microbiota, with microbiota regulation serving as a core component of its antiviral efficacy.	[346,347]
Quercetin	C_15_H_10_O_7_	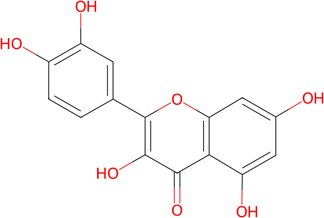	302.24	BALB/c mice MDBK cells	*in vivo* *in vitro*	Cp BVDV strain NADL Ncp BVDV strain BJ175170	Quercetin inhibits BVDV early infection by suppressing Hsp70 expression and modulates oxidative stress and ERK signaling (reducing p-ERK). It concurrently alleviates immunosuppression by elevating IFN-γ and IL-2 levels, thereby inhibiting viral replication and mitigating pathological damage.	[348]
Xanthohumol	C_21_H_22_O_5_	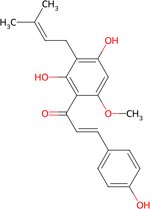	354.4	Primary calf testis cells	*in vitro*	BVDV strain NADL	Xanthohumol inhibits BVDV cytopathic effect, E2 protein expression, viral RNA synthesis. and modulating host lipid metabolism. It also acts synergistically with interferon α‑2b	[349,350]

Note: The chemical structures mentioned in the table are from https://www.chemspider.

### Effects on viral reproductive disorders in sheep

#### Effects of natural compounds against CpHV-1

Although natural products are gaining increasing attention in antiviral research, reports on their activity against CpHV-1 remain extremely scarce, with most studies limited to phenomenological observations. Only three articles are included in the discussion of this subsection. Ginger essential oil significantly and directly inactivates CpHV-1 virus particles [351]. *Ficus carica* latex demonstrates antiviral activity against CpHV-1, although its specific mechanism of action requires further investigation [352,353]. There are essentially no reports on the effects of other natural compounds against CpHV-1 beyond these few. This substantial gap in research severely limits the development of novel CpHV-1 intervention strategies based on natural products.

## Analysis of the common targets of natural compounds against different viruses

On the basis of a systematic analysis of the target sites of natural compounds against 11 animal viruses, the shared targets were summarized. Particular emphasis was placed on compounds that can act on the same target site in four or more viruses.

### Analysis of the antiviral commonalities of natural compounds targeting the NF-κB signaling pathway

**Figure 3.**
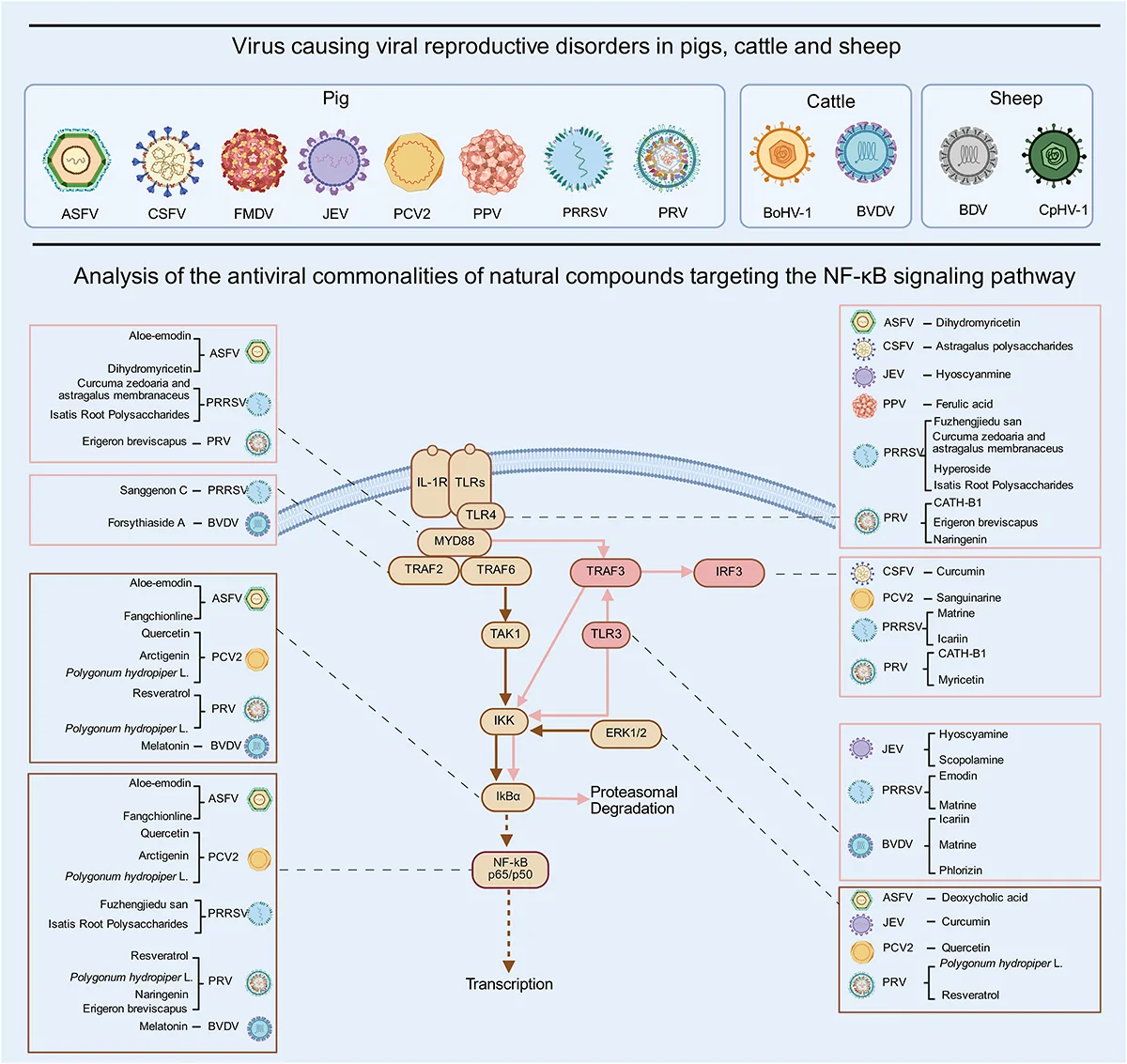
Schematic showing the mechanisms of action of natural compounds that target NF-κB pathway components. Key antiviral nodes within the NF-κB pathway. Critical intersections in the toll-like receptor (pink) and NF-κB (orange) signaling branches – including TLR4, IRF3, ERK1/2, IκBα, and p65 – mediate activity against four or more viruses. The symbols used are defined as follows: →: direct regulation; : indirect regulation; : direct inhibition; and : natural compounds with antiviral activity at the corresponding node, as well as their target viruses. Drawn by BioRender.

The NF-κB signaling pathway primarily regulates inflammation, immune responses, and cell survival. Under resting conditions, NF-κB remains sequestered in the cytoplasm through its interaction with the inhibitory protein IκB [354]. Upon viral infection, pathogens often hijack this pathway [355]. Excessive activation can establish a proinflammatory microenvironment that promotes viral replication, whereas insufficient activation helps the virus evade immune clearance [356]. The antiviral activity of natural compounds restores the normal function of the NF-κB pathway through precise regulation. The natural compounds discussed in this review act primarily on five core nodes: TLR4, IRF3, ERK1/2, IκBα, and p65. The following sections summarize their mechanisms of action and corresponding antiviral effects (Figure 3).

#### TLR4

TLR4 (mainly localized to the cell membrane), which is located primarily on the cell membrane, recognizes viral envelope proteins [357,358]. Upon the binding of these viral proteins to TLR4, downstream signaling is rapidly initiated via MyD88 and TIR-domain-containing adapter-inducing interferon-β (TRIF), leading to NF-κB activation [359]. In the case of viruses that weakly activate the NF-κB pathway to evade immune detection, such as PRV, certain natural compounds (such as CATH-B1) can upregulate TLR4 to moderately activate the immune response. In the case of viruses that overactivate the TLR4/NF‑κB pathway to establish a proinflammatory microenvironment (such as ASFV, CSFV, JEV, PPV, and PRRSV), natural compounds inhibit TLR4 expression, thereby disrupting the virus‑dependent inflammatory microenvironment, suppressing viral replication, and mitigating excessive inflammatory damage [360–362].

#### IRF3

IRF3 is not a direct component of the NF-κB complex but functions in synergy with it [363]. Upon the binding of viral or virus-derived double-stranded RNA to TLR3, TRIF is activated and recruits TNF receptor-associated factor 3, promoting the phosphorylation, dimerization, and nuclear translocation of IRF3 and thereby inducing type I interferon expression [364,365]. Six distinct natural compounds that exhibit antiviral activity against CSFV, PCV2, PRV, and BVDV exert their effects precisely by inducing IRF3 phosphorylation and nuclear translocation, thereby activating host antiviral and immunomodulatory responses.

#### IκBα

IκBα is a core inhibitory protein of NF-κB [366]. Upon activation by upstream signals, the inhibitor of nuclear factor kappa-B kinase complex is phosphorylated, leading to the degradation of IκBα and the subsequent release of the NF-κB dimer (p65/p50 complex) for nuclear translocation, where it initiates the transcription of pro-inflammatory genes [367]. Seven distinct natural compounds, each effective against one of the viruses ASFV, PCV2, PRV, and BVDV, exert their antiviral effects through the same target, inhibiting IκBα phosphorylation, thereby preventing NF‑κB nuclear translocation and subsequently suppressing the production of inflammatory mediators.

#### p65

p65 is the key transcriptional activation subunit of the NF-κB dimer (p65/p50), and its nuclear translocation is one of the final steps in NF-κB-mediated signaling [368]. Eleven natural compounds act against ASFV, PCV2, PRRSV, PRV, and BVDV by directly inhibiting the nuclear translocation of NF-κB p65. This intervention further suppresses inflammatory responses and enhances the overall antiviral response.

#### ERK1/2

ERK, a key component of the MAPK pathway, does not regulate NF-κB by influencing its nuclear translocation [369]. It increases transcriptional efficiency by phosphorylating intranuclear p65, thereby promoting the production of inflammatory cytokines [370]. Five distinct natural compounds that are effective against ASFV, JEV, PCV2, and PRV can inhibit ERK phosphorylation, thereby blocking its modification of p65. Even when NF‑κB is translocated to the nucleus, it cannot efficiently recruit coactivators, thereby significantly suppressing the transcription of downstream inflammatory genes and helping the host balance viral clearance with pathological damage.

Regulating upstream TLR4 can correct pathway dysregulation at the source and enable bidirectional modulation according to different viral strategies (overactivation or weak activation), thereby achieving immune rebalancing. Even if the virus bypasses upstream nodes via alternative pathways (such as IκBα degradation or ERK activation), blocking p65 nuclear translocation prevents full pathway activation. Moreover, directly targeting p65 may reduce nonspecific effects on other intracellular signaling pathways, thereby minimizing the potential side effects of natural compounds.

### Analysis of natural compound antiviral agents targeting autophagy

**Figure 4.**
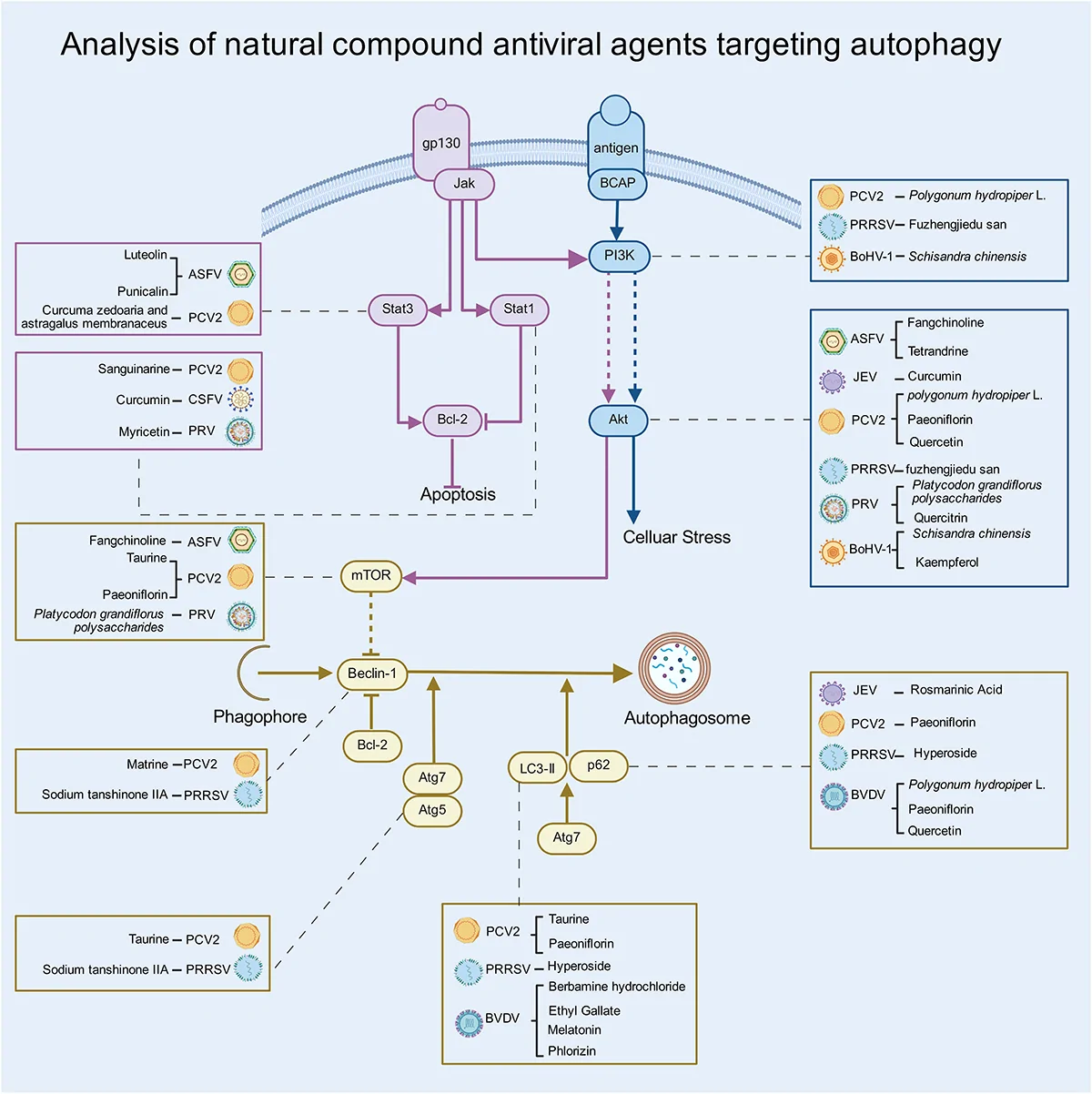
Schematic of the mechanisms through which natural compounds target autophagy. Key antiviral nodes within autophagy-associated pathways. Critical nodes in the jak–stat (purple), PI3K/AKT (blue), and autophagy (yellow) pathways, specifically AKT and p62, each mediate broad-spectrum activity against four or more viruses. The symbols used in the figure are defined as follows: →: direct regulation; : indirect regulation; : direct inhibition; and : natural compounds with antiviral activity at the corresponding node, as well as their target viruses. Drawn by BioRender.

Autophagy is a highly conserved intracellular catabolic pathway that maintains cellular function and homeostasis by degrading damaged or redundant cellular components through the lysosomal system [371]. During viral infection, the host can utilize selective autophagy to directly degrade viral components or restrict their spread [372]. However, some viruses (such as JEV) can induce autophagy to evade immunity and utilize autophagic vesicles as replication sites [373,374]. The natural compounds discussed in this review primarily target the key nodes AKT and p62. The following sections summarize their mechanisms of action and corresponding antiviral effects (Figure 4).

#### AKT

Upon binding to its receptor, the virus activates AKT via PI3K, which activates mTOR, increasing autophagic flux to clear viral components (such as dengue virus and herpesvirus infection) [373,375]. AKT also acts as a potent antiapoptotic signal, preventing cell death by inhibiting the activity of Caspase‑9 and others [376]. Several natural compounds and mixtures, including fangchinoline, Fuzhengjiedu San, kaempferol, curcumin, and quercetin, which exhibit activity against ASFV, JEV, PCV2, PRRSV, and BoHV-1, can attenuate Akt phosphorylation, increasing the susceptibility of infected cells to apoptosis and thereby eliminating the source of infection before the virus completes replication [377]. Interestingly, other compounds, such as paeoniflorin and *P. grandiflorus* polysaccharide, suppress PCV2- and PRV-induced autophagy by increasing the level of phosphorylated Akt, thereby restricting intracellular viral expansion. The different effects of natural compounds on the same virus may be attributed to differences in whether they act before or after the initiation of viral replication.

#### p62

p62 recognizes ubiquitinated substrates via its Ubiquitin-associated domain and recruits them to the autophagosome membrane through LC3, leading to aggregate formation and degradation [378,379]. Studies have shown that p62 can target and degrade the viral proteins of CSFV and BVDV [380,381]. Mechanistically, natural compounds with efficacy against PCV2, BVDV, and PRRSV – including paeoniflorin, hyperoside, and quercetin – can upregulate p62 expression, increasing the recognition and autophagic degradation of ubiquitinated viral particles and thereby directly limiting viral replication. The accumulation of p62 also activates inflammatory and immune signals such as NF‑κB, promoting the transcription of inflammatory cytokines and interferon-stimulated genes (ISGs) and thus establishing an antiviral state [379]. Conversely, rosmarinic acid, which has anti‑JEV activity, downregulates p62 expression. The underlying mechanism may involve hindering the clearance of abundant misfolded proteins generated during JEV replication, leading to the accumulation of toxic aggregates within viral factories while simultaneously exacerbating ER stress and inducing premature apoptosis of infected cells [137,382].

Natural compounds bidirectionally regulate host nodes such as AKT and p62: inhibiting AKT induces apoptosis of infected cells, whereas activating AKT restricts excessive autophagy. Upregulating p62 increases viral protein degradation and immune signaling, whereas downregulating p62 promotes viral aggregation and cell death. This multitarget, on-demand modulation clears viruses while avoiding excessive inflammatory damage, thereby rebalancing immunity.

### Analysis of natural compound antiviral agents targeting apoptosis

**Figure 5.**
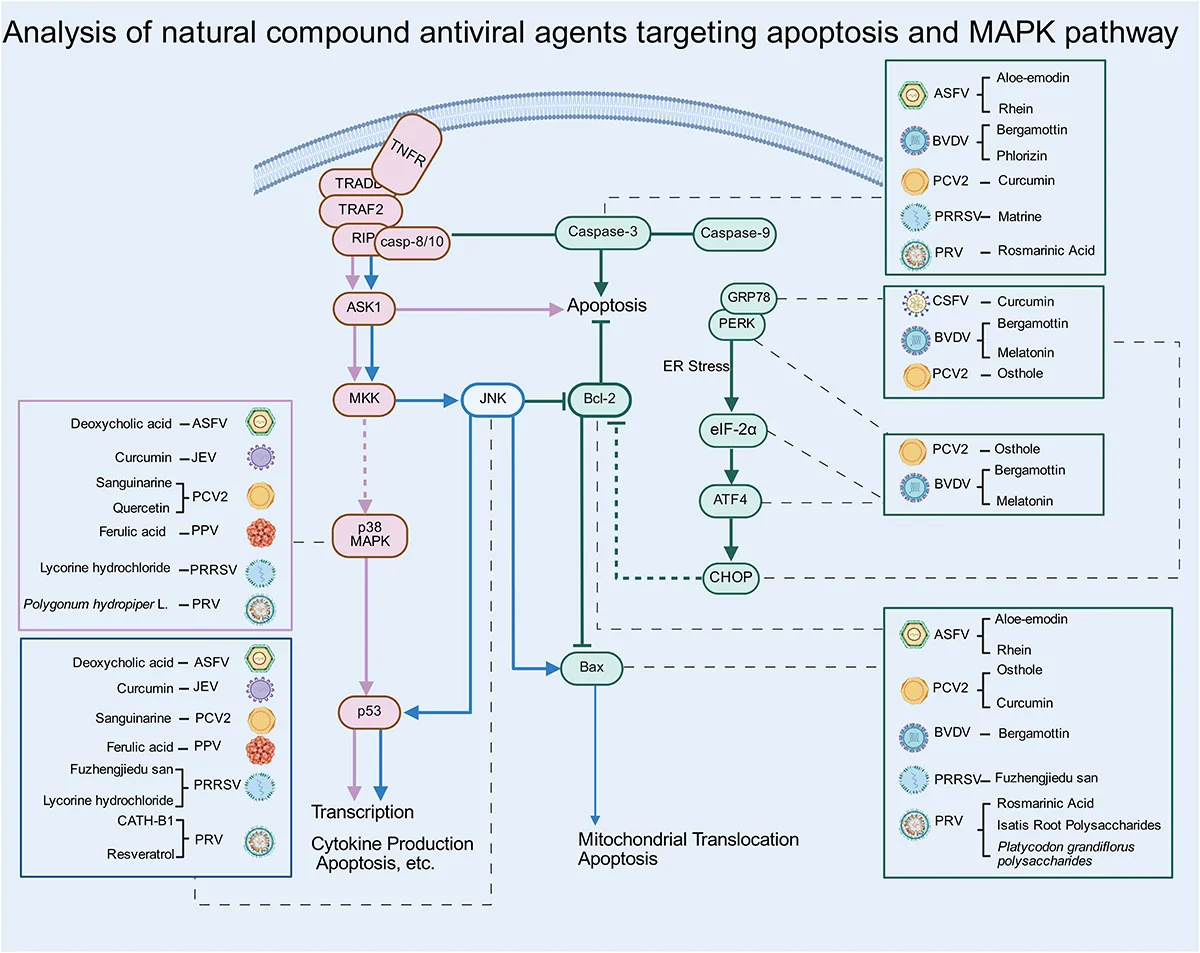
Schematic of the mechanisms through which natural compounds target apoptosis and the MAPK pathway. Key antiviral nodes in the apoptosis and MAPK pathways. Key hubs in the p38 MAPK (pink), SAPK/JNK (blue), and apoptosis (green) pathways – including p38 MAPK, JNK, caspase-3, Bcl-2, and Bax – demonstrate efficacy against four or more viruses causing reproductive disorders. The symbols used are defined as follows: →: direct regulation; : indirect regulation; : direct inhibition; : indirect inhibition; and : natural compounds with verified activity at the specified node, as well as their corresponding target viruses. Drawn by BioRender.

Apoptosis is a critically regulated process in cells that eliminates damaged or unnecessary cells to maintain tissue homeostasis [383]. The natural compounds discussed herein primarily target three core nodes, Caspase-3, Bcl-2, and Bax. In the following section, their mechanisms of action and corresponding antiviral effects are summarized in a point-by-point manner (Figure 5).

Viral infection often induces host apoptosis to eliminate infected cells and prevent viral spread [384]. This process typically relies on the activation of proapoptotic proteins such as Bax, leading to the release of mitochondrial cytochrome c, the subsequent activation of caspase-9 and, ultimately, the cleavage of caspase-3, thereby initiating apoptosis [385]. Antiapoptotic proteins such as Bcl-2 can inhibit this cascade and promote cell survival. ASFV relies on an intact cellular architecture for replication; thus, apoptosis can directly disrupt viral replication [386,387]. Natural compounds such as aloe-emodin and rhein activate Caspase-9, promote the formation of the Apaf-1/cytochrome c complex, and subsequently activate Caspase-3, thereby inducing macrophage apoptosis to eliminate infected cells and limit viral spread. On the other hand, natural compounds such as curcumin, bergamottin, and matrine, which are effective against PCV2, PRRSV, BVDV, and PRV, can suppress caspase-3 activity, upregulate antiapoptotic proteins such as Bcl-2, or downregulate proapoptotic proteins such as Bax. These actions help maintain mitochondrial membrane stability and prevent cytochrome c release, thereby protecting host cells from virus-induced apoptosis.

### Analysis of the antiviral commonality of natural compounds targeting the MAPK pathway

The MAPK signaling pathway regulates stress and inflammatory responses by phosphorylating downstream substrates [388]. The natural compounds summarized in this article act mainly on two core nodes: p38 MAPK and JNK. The following sections present their mechanisms of action and the corresponding antiviral effects associated with each node (Figure 5).

Following viral infection, the host activates mitogen‑activated protein kinase (MAPKKK, such as apoptosis signal‑regulating kinase 1) via pattern recognition receptors and adaptor proteins, which activates JNK and p38 MAPK through mitogen‑activated protein kinase ‑mediated phosphorylation [389,390]. Upon nuclear translocation, activated JNK/p38 stabilizes and activates p53, thereby inducing apoptosis of infected cells [391].

#### JNK

JNK is a key mediator of stress and inflammation [392]. Various natural compounds exhibit activity against multiple viruses, including ASFV, JEV, PCV2, PPV, PRRSV, and PRV, by targeting both the JNK and p38 MAPK nodes. Studies have indicated that PRV infection induces activation of the JNK pathway, which is characterized by a significant increase in JNK phosphorylation [393]. Although the natural peptide CATH‑B1 increases JNK phosphorylation, it inhibits PRV replication. This apparent paradox lies in the fact that CATH‑B1 reprograms JNK signaling into an antiviral response by modulating the spatiotemporal dynamics of the pathway (such as inducing perinuclear clustering) and its upstream mechanism (dependent on TLR4 and endosomal acidification), thereby redirecting virus‑activated JNK toward an antiviral outcome [279]. Sustained JNK activation promotes apoptosis (by phosphorylating Bax and inhibiting Bcl‑2) and induces the mitochondrial pathway of apoptosis. The antiviral effects of natural compounds with antiviral activity against ASFV, JEV, PCV2, PPV, and PRRSV are associated with the inhibition of JNK phosphorylation. The underlying mechanisms include alleviating JEV- and PRRSV-induced endoplasmic reticulum stress and subsequent apoptosis and neuroinflammation [394–396], disrupting the balance of JNK signaling necessary for PCV2 replication, thereby suppressing viral proliferation [397], and mitigating the excessive immunopathological damage caused by viral infection to protect host tissues.

#### p38 MAPK

p38 MAPK regulates the synthesis of proinflammatory cytokines such as TNF-α, IL-1β, and IL-6. Natural compounds that are effective against ASFV, JEV, PCV2, PPV, and PRV, such as curcumin, quercetin, and sanguinarine, can decrease p38 MAPK phosphorylation, thereby attenuating excessive inflammatory responses and directly creating an intracellular environment unfavorable for viral replication.

Natural compounds bidirectionally regulate JNK and p38 MAPK. Activating JNK reprograms it toward antiviral apoptosis; inhibiting JNK alleviates virus‑induced stress and immunopathology. Suppressing p38 MAPK attenuates excessive inflammation and creates an intracellular environment unfavorable for viral replication. This multitarget, on‑demand modulation clears a wide range of viruses (ASFV, JEV, PCV2, PPV, PRRSV, and PRV) while preventing collateral tissue damage, thereby re‑establishing host immune homeostasis and supporting broad‑spectrum antiviral therapy.

### Assessment of translational potential based on current evidence

To facilitate cross‑compound comparison, we compiled Table 8, which presents the available evidence for each lead compound individually. The information in this table was organized around three key dimensions: (i) the highest in vivo validation level achieved (target livestock species > rodent >in vitro only); (ii) mechanistic clarity; and (iii) class‑specific druggability limitations (summarized in Low druggability: chemical composition and pharmacokinetic drawbacks). Based on the evidence summarized in this table, we defined a four‑tier priority scale to reflect the translational readiness of each candidate: High = validated in target species (pig/cattle) with measurable efficacy; Medium = robust rodent data but lacking target‑animal validation; Low = only in vitro or adjuvant‑only evidence; Very Low/Negligible = extremely limited or no in vivo data.

**Table 8.** Summary of in vivo evidence and translational bottlenecks for lead compounds.

Virus	Lead Compounds	*In vivo* Validation Level	Primary Mechanism Target (Pathway*/*Action Type)	Key Translational Bottlenecks	Priority
ASFV	Genistein	Pig	ASFV topoisomerase II inhibition	Poor aqueous solubility/stability; low oral bioavailability; species-dependent metabolism; no residue/withdrawal data	High
	Resveratrol	*in vitro* only	Directly inhibits ASFV replication/antiviral; activates Nrf2 signaling pathway/antioxidant	Low oral bioavailability; extensive gut microbial transformation; dietary protein/mineral binding; PK data available in pigs (low bioavailability)	Medium
	Luteolin	*in vitro* only	Directly inhibits ASFV replication/Antiviral; suppression of NF‑κB, STAT3, and ATF6 pathways/anti-inflammatory, ER stress modulation	Poor aqueous solubility/stability; low oral bioavailability; PK data available in minipigs	Medium
CSFV	*Astragalus* polysaccharides	Pig (as vaccine adjuvant only)	E2 protein/Antiviral; TLR4 activation*/*immune enhancement	High molecular weight limits oral absorption; enzymatic/microbial degradation in gastrointestinal tract; high production cost; no direct antiviral efficacy in target species	Low
	Curcumin	*in vitro* only	FASN, ATF6, CHOP, GRP78/lipid metabolism interference; IRF3, IRF7, STAT1, ISG15/antiviral immune activation	Low oral bioavailability; extensive first-pass metabolism; PK data available in pigs (mainly with improved formulations)	Low
	Ginsenosides Rb2/Rb3	*in vitro* only	IRES/inhibition of translation initiation and viral protein synthesis	Poor oral absorption; hemolytic potential; PK data available (oral bioavailability low); no direct *in vivo* antiviral data in pigs	Low
FMDV	Mizoribine	Suckling mouse	IMPDH/inhibition of purine synthesis and early replication	Narrow therapeutic window; potential immunosuppression; no PK or efficacy data in pigs or cattle	Low
	Andrographolide	*in vitro* only	3Cpro (active site His46/Cys163, polyprotein processing) / inhibition of viral proteolytic maturation	High volatility; poor storage stability; emetic/hematological toxicity risk; PK data available (oral bioavailability low); no efficacy data in pigs or cattle	Low
	Apigenin	*in vitro* only	IRES/inhibition of IRES‑mediated protein translation	Poor aqueous solubility/stability; low oral bioavailability; PK data available in minipigs; no efficacy data in pigs or cattle	Low
JEV	Artemisinin	Mouse	inhibition of viral replication and virion production/antiviral	High volatility; poor storage stability; PK data available in pigs; no efficacy data in pigs	Medium
	Curcumin	*in vitro* only	NS5/antiviral; MAPK/anti‑apoptotic; UPS/proteasomal clearance	Low oral bioavailability; extensive gut microbial transformation; PK data available in pigs (mainly with improved formulations); no efficacy data in pigs	Medium
	Rosmarinic acid	Mouse	p62/ER stress‑dependent apoptosis	Low oral bioavailability; extensive gut microbial transformation; PK data available in pigs; no efficacy data in pigs	Medium
PCV2	Matrine	Mouse	STAT3/Beclin1 axis/antiviral replication; L. acidophilus/anti‑inflammatory and barrier maintenance	Hepatotoxicity risk; risk of drug interactions; lack of long term safety data in piglets	Medium
	Arctigenin	Mouse	direct inhibition of viral replication/antiviral; NF-κB/anti-inflammatory	Low oral bioavailability; extensive first pass metabolism; lacking piglet validation	Medium
	Epigallocatechin gallate	*in vitro* only	Capsid–heparan sulfate binding blockade	Extremely low oral bioavailability; extensive metabolism; strictly *in vitro* evidence only	Low
PPV	Ferulic acid	*in vitro* only	NS1/antiviral; NF‑κB/anti‑inflammatory; MAPK/anti‑apoptotic	Extremely limited studies; low oral bioavailability; no target species data	Very Low
	Propolis flavone	Britain White guinea pigs; Pig (as adjuvant only)	Inhibition of viral replication /Antiviral; Th1/Th2 humoral immunity enhancement	Adjuvant only; complex composition; poor batch to batch consistency	Very Low
PRRSV	Matrine	Pig	N protein*/*antiviral; autophagy regulation; HSPA8/HSP90AB1/ anti‑apoptotic; caspase‑3, NF‑κB/immunomodulatory	Hepatorenal toxicity risk; risk of drug interactions; narrow therapeutic window	High
	Xanthohumol	Pig	Viral entry/antiviral blockade/Nrf2 activation/ antioxidant; cytokines/anti‑inflammatory	Low oral bioavailability; poor solubility; rapid first pass metabolism	High
	Curcumin	*in vitro* only	endocytosis, membrane fusion, pH/inhibition of viral entry	Low oral bioavailability; poor solubility; rapid first pass metabolism; PK data available in pigs (mainly with improved formulations); no efficacy data in pigs	Medium
PRV	Resveratrol	Pig	OPN/antiviral; NF‑κB, IE180, cytokines/immunomodulatory; microglia/neuro; progesterone /endocrine	Low oral bioavailability; extensive first pass metabolism; gut microbial transformation; PK data available in pigs	High
	Curcumin	Rat	BDNF/neuroprotection; miR‑155 axis/antiviral	Low oral bioavailability; extensive first pass metabolism; gut microbial transformation; PK data available in pigs (mainly with improved formulations)	Medium
	Luteolin	Mouse	PRV mRNA, gB/antiviral; SOD, GSH/antioxidant; IFN/immunomodulatory	Poor aqueous solubility/stability; low oral bioavailability; extensive first pass metabolism; PK data available in minipigs	Medium
BoHV-1	Curcumin	*in vitro* only	Lipid raft*/*viral entry interference	No *in vivo* validation in cattle; low oral bioavailability; rumen degradation risk; PK data available in pigs (not cattle)	Very Low
	Kaempferol	*in vitro* only	PI3K*/*Akt signaling inhibition	No *in vivo* validation in cattle; poor aqueous solubility/stability; low oral bioavailability	Very Low
BVDV	Melatonin	Mouse	ER stress, LC3‑II*/*autophagy regulation; NF‑κB/anti‑inflammatory; antiviral factors, tight junctions/antiviral	Rapid metabolic inactivation; short half-life; rumen degradation risk; no PK or efficacy data in cattle	Medium
	Quercetin	Mouse	Hsp70/Antiviral; ERK, cytokines/immunomodulatory	Poor aqueous solubility/stability; low oral bioavailability; rumen degradation risk; PK data available in pigs; no PK or efficacy data in cattle	Medium
	Phlorizin	Mouse	Beclin-1, LC3B-II/autophagy; inhibition of viral replication/antiviral; modulation of IFN-I, RIG-I, TLR3, NLRP3, T-cell ratios, and microbiota/immunomodulatory	Poor oral absorption; low oral bioavailability; rumen degradation risk; PK data available (not cattle); no PK or efficacy data in cattle	Medium
CpHV-1	Ginger essential oil	*in vitro* only	Inactivates CpHV-1 virus particles/antiviral	Highly volatile; unstable during feed processing; hepatorenal toxicity risk; pungent taste reduces feed intake; only one report	Negligible

The resulting ranking shows that only a handful of candidates – genistein, matrine, xanthohumol, and resveratrol – have been validated in pigs with measurable efficacy, whereas the vast majority remain at the rodent or *in vitro* stage. For these top‑ranked leads, the next logical step is to prioritize formulation development (to overcome poor bioavailability and metabolic barriers) and regulatory‑grade safety assessments in target species, rather than additional in vitro mechanistic studies. These findings provide a pragmatic, evidence-based reference for prioritizing translational investment and avoiding low yield screening of crude extracts with limited clinical potential.

While our analysis reveals common host targets (NF-κB, MAPK, apoptosis, and autophagy), these pathways are primarily involved in inflammation and cell survival. Compounds modulating these nodes may reduce virus-induced immunopathology but do not necessarily eliminate the virus. Therefore, we advocate that future studies should differentiate between antiviral efficacy (viral load reduction) and anti-inflammatory efficacy (cytokine suppression) in their readouts, and prioritize compounds with both activities.

## Research limitations of natural compounds for antiviral use in livestock

Pigs, cattle, and sheep are the core livestock species used in animal husbandry, and their reproductive performance directly affects the economic benefits of farming. This review summarizes the antiviral activities and mechanisms of various natural compounds against different viruses associated with reproductive disorders in livestock. Overall, the current application of natural compounds as antiviral agents has two major limitations: a translational gap and poor druggability. The former involves the limitations of purely *in vitro* studies and the lack of comprehensive clinical evaluations, whereas the latter pertains to the intrinsic physicochemical and pharmacokinetic flaws of the compounds themselves. In addition, species-specific obstacles exist for different farm animals.

### *Translational gap*: in vitro *constraints and lack of clinical data*

*In vitro* limitations More than two-thirds of the studies reviewed are purely *in vitro* experiments, with only a few including *in vivo* investigations, a considerable proportion of which have employed non-target animal models. This imbalance arises because challenging models involve biosafety risks, making them difficult to implement under actual farm conditions. Moreover, significant interspecies differences in drug metabolism, immune responses, and physiological structures prevent direct extrapolation of laboratory findings to pigs, cattle, and sheep. Notably, the control of infectious diseases in livestock often relies on herd-based treatment strategies, whereas most experimental designs use gavage or individual oral administration. The applicability of such approaches to herd-level delivery remains unverified.

Missing clinical evaluation. Even when *in vivo* validation has been carried out in target species, studies commonly suffer from small sample sizes, the use of challenge models rather than natural infection, short observation periods, and a lack of dose-finding and long-term safety assessments. Consequently, their results poorly reflect the protective efficacy under real production conditions. In addition, these *in vivo* trials largely report only short-term endpoints, such as viral loads or antibody titers, and systematically neglect key long-term clinical parameters, including feed intake, growth performance, morbidity, and mortality.

### Low druggability: chemical composition and pharmacokinetic drawbacks

Most investigations are confined to crude extracts or unrefined formulas, leaving the active constituents and their effective doses undefined. Batch-to-batch quality varies because of differences in species, origin, harvest season, and extraction procedures, resulting in unstable efficacy. Such mixtures may contain hepatotoxic, nephrotoxic, reproductive/developmental toxic, or sensitizing substances, but the absence of compound‑level identification precludes the attribution of toxicity. Polysaccharides and peptides have poor oral absorption, are readily inactivated, and incur high production costs. Marine products suffer from low purity, scarce content, and a lack of industry standards and regulatory oversight. For small‑molecule natural monomers (flavonoids, polyphenols and so forth), common shortcomings include low oral bioavailability, poor solubility, or pronounced systemic toxicity. Examples include the hepatotoxicity and pseudoaldosteronism of glycyrrhizic acid and the cardiotoxicity of matrine and emetine, with emetine also inducing vomiting. Fungal metabolites display narrow therapeutic windows and poor batch‑to‑batch consistency.

### Species-specific application barriers in livestock

Ruminal degradation is a major challenge for ruminants. Polysaccharides, flavonoids, alkaloids, and peptides are readily metabolized and inactivated by rumen microorganisms, rendering oral administration virtually ineffective. Rumen‑bypass formulations (coatings or liposomes) are needed, but these markedly increase the technical difficulty and cost. In pigs, the bitter or irritating taste of many herbal compounds reduces feed intake, particularly in weaned piglets. Long‑term supplementation may also induce gut dysbiosis. Furthermore, for all natural compounds used in food‑producing animals, there is a potential risk of tissue residue accumulation in the food chain following prolonged use. However, current evaluations addressing this risk are almost entirely absent.

Furthermore, the current regulatory framework for natural compounds in veterinary medicine, including drug registration, maximum residue limits, and safety evaluation guidelines, remains underdeveloped. Moving forward, collaborative efforts between industry regulators and the research community are urgently needed to establish and harmonize these standards. In addition to the compound class specific limitations discussed above, drug–drug interactions – particularly for alkaloids that may inhibit or induce CYP450 enzymes – remain largely unexplored but could compromise the safety and efficacy of co administered veterinary therapeutics (e.g. antimicrobials, antivirals). This risk, together with the systemic gaps in pharmacokinetics, regulatory frameworks, and food safety, reinforces the urgent need for target species translational studies rather than further in vitro screening.

## Summary and outlook

Compared with conventional synthetic compounds, natural compounds possess several unique advantages in antiviral applications. They are widely available, highly biodegradable, and have short environmental persistence; thus, they impose a lower burden on ecosystems than most synthetic drugs do. Many natural products exhibit multitarget characteristics and exert integrated effects by simultaneously modulating viral replication, host immunity, and inflammatory pathways, which makes it difficult for viruses to develop drug resistance. In contrast, chemical drugs often rely on a single target and carry a relatively high risk of resistance. Certain essential oils with antiviral activity and their volatile components can disperse in the air, theoretically limiting the aerosol transmission of viruses in confined livestock houses, thereby providing an environment‑mediated auxiliary control measure.

Notably, extensive data on the formulation technology, clinical safety, toxicity profiles and efficacy of many natural medicines in human medicine have already been obtained. This data provides a valuable foundation for their repurposing in veterinary medicine. Human and veterinary drugs differ in target species, routes of administration, and residue requirements; however, the research and development cycle can be substantially shortened, and costs can be reduced through rational dosage form adjustments (such as rumen‑bypass coating), the optimization of dosing regimens, and supplementary safety evaluations in target animals. Therefore, fully exploiting the veterinary potential of human‑used natural medicines is important for overcoming the current deficiencies of natural compounds in livestock applications.

The broad‑spectrum regulatory effects of natural compounds on host responses stem from their selective targeting of conserved host nodes, but these effects should not be equated with direct antiviral activity. Analysis of the shared mechanisms of action of different natural compounds revealed that their targets include proteins in the NF‑κB pathway (TLR4, p65, IRF3, IκBα, and ERK1/2), the MAPK signaling pathway (p38 MAPK and JNK), the apoptosis axis (Caspase‑3, Bcl‑2, and Bax), and the PI3K-AKT-mTOR autophagy network (AKT and p62). Acting on host-conserved targets enables the inhibition of multiple viruses by modulating shared host pathways and, owing to the evolutionary conservation of host proteins, significantly reduces the risk of resistance caused by viral mutations. However, given that most of these compounds primarily target host inflammatory pathways rather than viral components, they are best positioned as adjuncts to existing antiviral drugs and vaccines rather than as independent therapies. Future research may also focus on the domain interactions and conformational changes of key targets (TLR4, p65, AKT, and JNK), quantify the dependence of different viruses on the NF‑κB pathway, and develop multivirus coinfection models to screen for natural compound combinations that synchronously regulate JNK-AKT crosstalk and balance autophagy and apoptosis, thereby providing broad‑spectrum or virus‑specific antiviral therapies with high efficacy and low toxicity.

**Figure 6.**
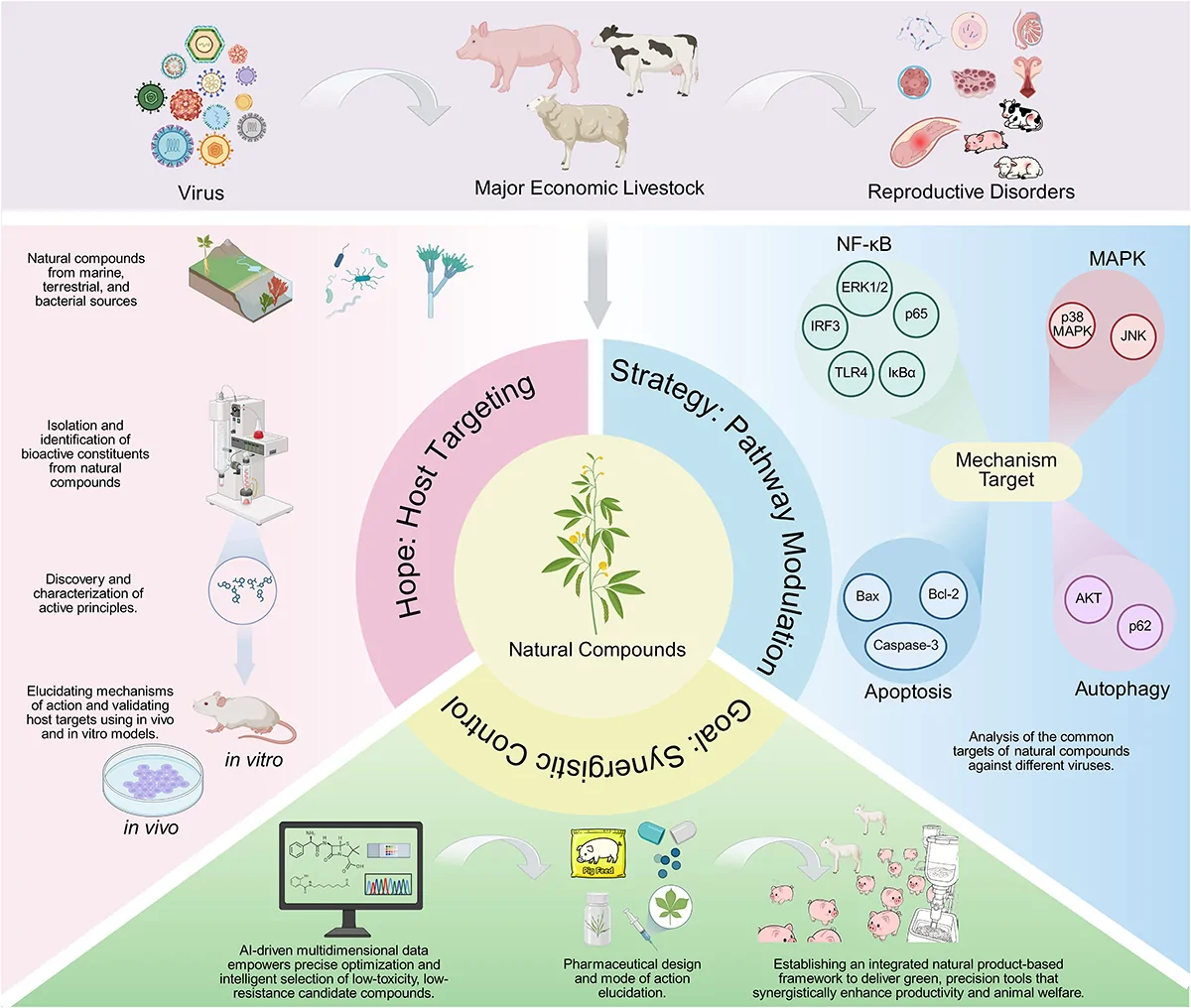
Summary of the integration of natural compounds and host targeting strategies to achieve broad-spectrum antiviral resistance in livestock. Virus-induced reproductive disorders represent a considerable impediment to the sustainable development of the livestock industry. In contrast to conventional antiviral agents, whose use is often limited because of drug resistance, natural compounds demonstrate broad-spectrum antiviral potential through the modulation of conserved host signaling pathways, offering safer and multitargeted therapeutic alternatives. However, current research remains largely limited to cell-level studies, and extensive natural resources have yet to be systematically explored. Future investigations should prioritize the large-scale screening and synergistic pairing of natural compound constituents, develop coinfection models, and employ integrated AI and multiomics technologies to design multitarget formulations. The incorporation of these compounds into existing disease management frameworks via AI-assisted strategies is expected to facilitate the development of more sustainable, precise, and environmentally sound disease control approaches. Drawn by BioRender.

Finally, natural compounds should not be regarded as standalone replacements for vaccines or conventional antivirals but rather as adjuncts that complement existing disease prevention and control strategies. For example, they can be administered during vaccination campaigns to mitigate vaccine‑induced inflammatory side effects, or used as feed additives to reduce viral shedding during outbreaks, thereby reinforcing culling and biosecurity measures. With the aid of artificial intelligence-assisted structure-activity optimization, multiscale joint studies and database construction, the development of standardized formulations and administration protocols can be promoted. The overall goal is to provide green, precise, and sustainable natural-origin antiviral tools against major reproductive-disorder viruses in pigs, cattle, and sheep, improving both productivity and animal welfare (Figure 6).

## Supplementary Material

Table S2 Literature review results by database and search query.xlsx

Table S1 List of abbreviations used in this study.docx

File S1 PRISMA 2020 Checklist for Systematic Reviews.docx

legends for supplementary file and tables.docx

## Data Availability

The data that support the findings of this study are openly available in Science Data Bank at https://doi.org/10.57760/sciencedb.45848 [398]
