# Supplementary material for: The roles and mechanisms of natural compounds in preventing and treating viral reproductive disorders in major economic livestock
Source: Virulence. 2026 Aug 24;17(1):2723577. doi: 10.1080/21505594.2026.2723577 (PMC13532028; doi:10.1080/21505594.2026.2723577)
Supplement: Supplementary file 1 [file KVIR_A_2723577_SM4976.docx]

**legends for supplementary file and tables**

This page provides the captions/legends for all supplementary files and tables associated with this manuscript. No supplementary figures are included. Supplemental data for this article can be accessed online at https://doi.org/10.57760/sciencedb.45848

**File S1 |** The PRISMA 2020 Checklist

PRISMA 2020 checklist for this review. This document details how the review adheres to the 27‑item PRISMA 2020 standard across the title, abstract, introduction, methods, results, discussion, and funding sections

**Table S1 |** List of abbreviations used in this study

A comprehensive list of all abbreviations used in this study, presented in alphabetical order alongside their corresponding full terms. This table is intended to aid readers in navigating the technical terminology and acronyms employed throughout the manuscript.

**Table S2 |** Literature review results by database and search query

Detailed documentation of the systematic search strategy. This table presents the virus-specific search queries adapted for database, along with the number of records initially identified per query and per database prior to any deduplication or screening.
