# Supplementary material for: The roles and mechanisms of natural compounds in preventing and treating viral reproductive disorders in major economic livestock
Source: Virulence. 2026 Aug 24;17(1):2723577. doi: 10.1080/21505594.2026.2723577 (PMC13532028; doi:10.1080/21505594.2026.2723577)
Supplement: Supplementary file 3 [file KVIR_A_2723577_SM4978.docx]

| **Abbreviation** | **Definition** |
| --- | --- |
| 3Cpro | 3C protease |
| 3Dpol | 3D polymerase |
| *A. indica* | *Azadirachta indica* |
| *A. macrocephala* | *Atractylodes macrocephala* |
| *A. membranaceus* | *Astragalus membranaceus* |
| *A. Radix* | *Astragali Radix* |
| Akt | Ak strain transforming |
| ASFV | African swine fever virus |
| ATF6 | Activating transcription factor 6 |
| ATP | adenosine triphosphate |
| Bax | BCL2-associated X |
| Bcl-2 | B-cell lymphoma-2 |
| BDV | Border disease virus |
| BoHV-1 | Bovine herpesvirus type 1 |
| BVDV | Bovine viral diarrhea virus |
| *C. dactylon* | *Cynodon dactylon* |
| *C. militaris* | *Cordyceps militaris* |
| *C. phaeocaulis* | *Curcuma phaeocaulis* |
| *C. pilosula* | *Codonopsis pilosula* |
| *C. sappan* | *Caesalpinia sappan* |
| *C. triandra* | *Tiliacora triandra* |
| *C. volvatus* | *Cryptoporus volvatus* |
| CATH-B1 | Cathelicidin-B1 |
| CD28 | Cluster of differentiation 28 |
| CHOP | C/EBP-homologous protein |
| CpHV-1 | Caprine herpesvirus type 1 |
| CSFV | Classical swine fever virus |
| *E. breviscapus* | *Erigeron breviscapus* |
| *E. globulus* | *Eucalyptus globulus* |
| ERK1/2 | Extracellular signal‑regulated kinase 1/2 |
| FASN | Fatty acid synthase |
| FMDV | Foot-and-mouth disease virus |
| GAS | Gamma-interferon activation sequence |
| *G. lucidum* | *Ganoderma lucidum* |
| *G. skottsbergii* | *Gigartina skottsbergii* |
| GRP78 | Glucose regulated protein 78 |
| GSDMD-N | N-terminal domain of gasdermin D |
| GSH | Glutathione |
| *H. cordata* | *Houttuynia cordata* |
| *H. rhamnoides* | *Hippophae rhamnoides* |
| HAT | Histone acetyltransferase |
| HDAC | Histone deacetylase |
| *I. indigotica* | *Isatis indigotica* |
| IFN-α/β | Type I interferon-alpha/beta |
| IFN-γ | Interferon-gamma |
| IL-2 | Interleukin-2 |
| IL-6 | Interleukin-6 |
| IL-8 | Interleukin-8 |
| IMPDH | Inosine-5′-monophosphate dehydrogenase |
| iNOS | Inducible nitric oxide synthase |
| IRF3 | Interferon regulatory factor 3 |
| IRF7 | Interferon regulatory factor 7 |
| ISG15 | Interferon-stimulated gene 15 |
| IκBα | Inhibitor of nuclear factor kappa B alpha |
| JAK | Janus kinase |
| JEV | Japanese encephalitis virus |
| JNK | C-Jun N-terminal kinase |
| *L. christinae* | *Lysimachia christinae* |
| *L. edodes* | *Lentinula edodes* |
| *L. latifolia* | *Lavandula latifolia* |
| *M. nitidum* | *Monostroma nitidum* |
| *M. oleifera* | *Moringa oleifera* |
| MAPK | Mitogen-activated protein kinase |
| MCP-1 | Monocyte Chemoattractant Protein-1 |
| MDA | Malondialdehyde |
| mTOR | Mechanistic target of rapamycin |
| MX1 | MX dynamin-like GTPase |
| MYD88 | Myeloid differentiation primary response gene 88 |
| NF-κB | Nuclear factor kappa-B |
| NLRP3 | NOD-like receptor thermal protein domain associated protein 3 |
| *P. alliacea* | *Petiveria alliacea* |
| *P. australis* | *Phragmites australis* |
| *P. grandiflorus* | *Platycodon grandiflorus* |
| *P. hydropiper L.* | *Polygonum hydropiper L.* |
| *P. notoginseng* | *Panax notoginseng* |
| *P. sylvestris* | *Pinus sylvestris* |
| p53 | Tumor protein p53 |
| p65 | Nuclear factor kappa-B subunit p65 |
| PCV2 | Porcine circovirus type 2 |
| PI3K/Akt | Phosphatidylinositol 3-kinase/protein kinase B |
| PPV | Porcine parvovirus |
| PRRSV | Porcine reproductive and respiratory syndrome virus |
| PRV | Pseudorabies virus |
| *Q. brasiliensis* | *Quillaja brasiliensis* |
| *R. crenulata* | *Rhodiola crenulata* |
| *R. glutinosa* | *Rehmannia glutinosa* |
| RdRp | RNA-dependent RNA polymerase |
| ROS | Reactive Oxygen Species |
| *S. officinalis* | *Salvia officinalis* |
| *S. suberectus* | *Spatholobus suberectus* |
| *S. veitchii* | *Sasa veitchii* |
| SOD | Superoxide dismutase |
| STAT1 | Signal transducer and activator of transcription 1 |
| STAT3 | Signal transducer and activator of transcription 3 |
| STAT | Signal transducer and activator of transcription |
| *T. catigua* | *Trichilia catigua* |
| *T. canadense* | *Desmodium canadense* |
| *T. mongolicum* | *Taraxacum mongolicum* |
| TGF-β | Transforming growth factor-β |
| Th1/Th2 | T helper 1 cell / T helper 2 cell |
| TLR4 | Toll-like receptor 4 |
| TMAO | Trimethylamine N-oxide |
| TNF | Tumor necrosis factor |
| TNF‑α | Tumor necrosis factor‑alpha |
| TRIF | TIR-domain-containing adapter-inducing interferon-β |
